# Mental health interventions performed by nurses in Primary Health Care: analysis according to professional profile*

**DOI:** 10.1590/1980-220X-REEUSP-2025-0463en

**Published:** 2026-07-20

**Authors:** Rafaela Sales Medeiros, Divane de Vargas, Jaqueline Lemos de Oliveira

**Affiliations:** 1 Universidade de São Paulo, Escola de Enfermagem, São Paulo, SP, Brazil.

**Keywords:** Nurses, Mental Health Assistance, Primary Health Care

## Abstract

**Objective:**

To identify the variables related to nurses’ perception of recognizing mental health needs and implementing interventions in the context of Primary Health Care.

**Method:**

A cross-sectional study was conducted with 239 nurses from Primary Health Care. The identified interventions were grouped into three components: universal interventions; interventions specific to mental health and psychiatry; and educational and managerial interventions. Participants assessed the feasibility of implementation, its relevance to their scope of work, and their self-perception of capability to perform the interventions, responding to a structured instrument that underwent content review by experts and a pilot test, which showed satisfactory internal consistency (α = 0.77–0.90). Cluster analysis was used to identify distinct professional profiles regarding the perception of attribution, feasibility, capability, and frequency of performance of the interventions. Subsequently, log-binomial regression was applied to analyze the association between the variables of interest and the identified clusters.

**Results:**

Among the participants, 48.4% reported daily care for mental health demands, with anxiety being the most prominent (71.1%). Cluster analysis indicated that younger professionals and those with specific training in chemical dependency had higher scores in all investigated components. The component related to specific interventions in mental health had the lowest scores and perception of competence.

**Conclusion:**

It is necessary to increase investments in the training and capacity building of nurses to carry out mental health interventions in Primary Health Care.

## INTRODUCTION

Mental disorders comprise a heterogeneous group of conditions that vary according to severity, course, and functional impact. The literature has increasingly distinguished common mental disorders (CMDs) from severe mental disorders (SMDs). CMDs, such as mild to moderate depression, anxiety, and somatoform complaints, are highly prevalent in Primary Health Care (PHC) and are associated with significant psychological distress, although they generally do not imply severe impairment of autonomy or disruption of social relationships. In contrast, SMDs, including schizophrenia, persistent psychotic disorders, bipolar disorder, and severe substance use disorders, require continuous follow-up, more complex care, and close coordination with Psychosocial Care Network (RAPS - *Rede de Atenção Psicossocial*) services. It is estimated that eight out of ten people with SMDs do not receive treatment^(1)^. The combination of high prevalence and low identification reinforces the urgency of structured responses within the healthcare system.

In this context, the integration of mental health into PHC has been identified as a key strategy for reducing these problems within communities^(2)^. Due to its capillarity and close relationship with local territories, PHC can expand access, promote early detection, and provide timely interventions to reduce the invisibility and undertreatment of mental health problems. Nevertheless, integrating mental healthcare into PHC still faces several challenges, particularly in resource-limited settings. The lack of trained professionals, insufficient funding, and weaknesses in continuing education are recurrent barriers. In addition, the persistent lack of awareness and adequate training among physicians, nurses, and community health workers contributes to stigma and discrimination surrounding mental disorders^(3,4)^.

Actions performed by non-specialists can expand the capacity of frontline mental healthcare services and significantly improve care. In this context, especially in low- and middle-income countries such as Brazil, where access to specialized professionals is limited, nurses constitute important resources for addressing these problems. PHC nurses are often the first point of contact between users and the healthcare system. Beyond physical care, they play a central role in mental healthcare, particularly in establishing therapeutic bonds, qualified listening, and therapeutic communication. These dimensions of mental healthcare enable nurses to develop interventions for crisis management and the early identification of signs of CMDs and SMDs. Thus, they contribute not only to improving service capacity in dealing with mental health conditions, but also to coordination with the RAPS^(5,6)^.

However, despite the potential of these professionals in addressing this issue, they still face difficulties in expanding their scope of mental health actions within PHC^(7)^. The scarcity of professional training, the limited attention devoted to this issue during undergraduate education^(8,9)^, and stigma toward mental illness^(3)^ contribute to the low identification and use of mental health nursing interventions as an inherent practice within the PHC setting^(7)^.

Although the barriers faced by nurses in implementing mental health actions in PHC are well documented^(7,8,9)^, these findings are often investigated based on the workers’ own perceptions. However, the literature indicates that perceived competence does not always correspond to actual performance in clinical practice. Self-efficacy theory argues that an individual’s belief in their ability to perform certain actions influences their behavior and decision-making; nevertheless, such perception may not necessarily reflect the actual level of competence or the quality of care practices performed^(10,11)^. In this regard, studies in education and health professions have discussed the existence of a gap between self-perceived skills and observed performance, referred to as the risk of “illusion of competence”^(10,11,12)^. This reinforces the importance of competency-based approaches that consider not only professionals’ confidence, but also the effective mobilization of knowledge, skills, and attitudes in clinical practice.

No published studies were identified that allow the identification of distinct profiles of nurses according to sociodemographic, educational, and training characteristics that reveal groups with greater or lesser propensity to incorporate mental health actions into their daily PHC practice. Therefore, investigations capable of identifying possible differences among specific groups advance previous research, which generally describes practices in an aggregated manner without distinguishing internal patterns of professional heterogeneity. An analysis considering these aspects enables the development of more precise educational and managerial strategies aligned with the specific needs of each professional profile, thereby strengthening the institutionalization of mental health nursing care within PHC practice.

Given this scenario, understanding the factors that influence nurses’ performance, considering educational, institutional, and subjective aspects that determine their ability to identify and implement such interventions, becomes important. Seeking to answer the question, “Which variables (sociodemographic, educational, and/or institutional) are associated with greater identification and implementation of mental health interventions by nurses working in PHC?”, this study aimed to identify the variables related to nurses’ perceptions regarding the recognition of mental health demands and the implementation of interventions in the PHC context.

## METHOD

### Study Design

This was a cross-sectional study based on the STrengthening the Reporting of OBservational studies in Epidemiology guidelines.

### Setting and Data Collection Period

According to the Municipal Health Department, at the time of data collection, the municipality had 468 Basic Health Units (BHUs). Of these, 81 were hybrid units associated with specialty outpatient services and were excluded from the study, resulting in a total of 387 traditional BHUs included in the study. All managers of the potentially eligible units were individually contacted by e-mail and telephone to clarify the study objectives and request authorization for data collection. Ultimately, 66 (17%) BHUs agreed to participate and comprised the study setting. Data collection took place between March and August 2023.

### Sample

The sample consisted of nurses working in the participating units. The inclusion criteria were: a) having been employed at the institution for at least six months, a period considered sufficient to ensure operational experience within the territory and exposure to mental healthcare routines; and b) having provided at least one mental health consultation, defined as any consultation, reception, or referral related to psychological complaints, signs of mental distress, problematic substance use, or follow-up of individuals with CMDs or SMDs. Considering the total number of nurses employed by the Municipal Health Department and allocated to the participating BHUs at the time of data collection (N = 403), the sample size calculation adopted a 5% margin of error, an estimated refusal and loss rate of 5%, the inclusion of 20% to control for potential confounding factors, and a 95% confidence level, resulting in a minimum estimated sample of 207 participants. At the end of data collection, 239 nurses met the inclusion criteria and all agreed to participate in the study. To ensure territorial representativeness and precision of the estimates, stratified sampling proportional to the number of nurses in each administrative region was adopted. The final distribution of participants included eight nurses from the Central region, 69 from the South region, 19 from the West region, 38 from the North region, and 73 from the East region. This strategy ensured balanced inclusion of the different territories and reflected the organizational and demographic heterogeneity of the PHC network in the municipality of São Paulo.

### Data Collection

Data were collected using a questionnaire developed by the authors, containing 43 questions distributed into three sections: Section I – included 16 variables investigating issues related to professional profile, academic education, and specific training in mental health, in addition to sociodemographic variables such as sex, age, marital status, characterization of the educational institution (public, private, or other), time since graduation (in months/years), and preparation to work in mental health and/or substance dependence during undergraduate education. This section sought to identify whether the content had been predominantly offered through lectures, seminars, instructor-guided discussions, or extracurricular activities; Section II – addressed the profile of specific training in mental health and substance dependence care and included seven variables investigating whether the professional had completed graduate education and received training in mental health or substance dependence after graduation, as well as the frequency of care provided for the main mental health conditions (depression, anxiety, schizophrenia, and somatic symptoms related to psychological distress); Section III – consisted of 20 mental health nursing interventions in PHC described in national and municipal regulations and technical documents, including the Primary Care Booklets of the Ministry of Health, the Primary Care Guidelines of the Municipal Health Department of São Paulo (*Diretrizes de Atenção Básica da Secretaria Municipal de Saúde de São Paulo*)^(13,14)^, and COFEN Resolution 599/2018^(15)^, which address nursing practice in mental health. Additionally, interventions identified in an evidence review on mental health nursing practices in PHC^(7)^ were also selected. These interventions were grouped, according to the Primary Care Guidelines of the Municipal Health Department of São Paulo^(14)^, into three components: Component I – Universal practice interventions, including interventions that interface with other PHC care lines; Component II – Specific mental health and psychiatric practice interventions; and Component III – Educational and managerial interventions, related to preparing the nursing team to address mental health situations and nursing documentation (Chart 1).

**Chart 1:** Distribution of interventions according to the classification in the three intervention components – São Paulo, SP, Brazil, 2023.

Component I interventions	Component II interventions	Component III interventions
1. Welcoming	3. Counseling	16. Health education
2. Screening	5. Therapeutic relationship	17. Continuing education
4. Nursing consultation	7. Patient safety related to the use of psychotropic drugs	20. Nursing record
6. Prenatal care	8. Referrals/matrix support	
10. Home care	9. Social support	
15. Integrative and complementary practices	11. Family support	
19. Case management	12. Individual Therapeutic Project	
	13. Therapeutic groups	
	14. Applying scales	
	18. Advanced practices in psychosocial rehabilitation	

Although the interventions were extracted from normative and scientific sources that already recognized them as valid and recommended practices within the national context, an expert panel assessment was conducted to verify the adequacy of allocating these interventions into the three proposed components^(14)^. Five experts participated in this process, two with experience in PHC and three in mental health. The specialists analyzed the relevance and adequacy of each of the 20 interventions in relation to their respective components, also considering whether these interventions were aligned with the scope of nursing actions performed in healthcare services. This procedure aimed to ensure greater coherence between the interventions and the adopted analytical components. Subsequently, before definitive data collection, a pilot test was conducted by administering the instrument to eight nurses belonging to the target population of the study. This procedure aimed to assess question clarity, comprehension, and adequacy in relation to the proposed dimensions. The pilot test also made it possible to verify questionnaire structure consistency, the average completion time, and participants’ understanding of items and response scales, allowing minor adjustments before final application of the instrument. The results of these phases demonstrated good understanding of items and adequate allocation of the interventions within the proposed components, and no additional modifications were required before final application.

For each intervention listed in the three components, participants were asked about the frequency of performance of the intervention in daily practice, whether they recognized it as part of PHC nurses’ role, and their perception of preparedness to perform it. To create the nine scores corresponding to components I, II, and III, a score of 3 was assigned to “yes” responses, 2 to “not sure”, and 1 to “no” for the questions: “Do you consider this intervention feasible to perform at the BHU?” and “In your opinion, is this intervention part of PHC nurses’ role?” For the question “Do you feel prepared to perform this intervention?”, a score of 3 was assigned to “fully”, 2 to “partially”, and 1 to “unable”. Considering that data collection occurred during nurses’ working hours, a three-point Likert scale was adopted to facilitate understanding, reduce response time, minimize ambiguities, and promote greater consistency in responses. For interpretation purposes, the scores for each question, considering each component, were calculated to generate a score ranging from 0 to 100, with higher scores indicating better outcomes. Considering responses to each item as a 3-point Likert scale, the process began with calculating the raw score (RS) of the instrument. The RS was obtained by averaging the item scores within each dimension (Chart 1).

This calculation was performed using the following formula:


$$EB=\frac{q1+q2+\cdots +qn}{n}$$

Based on the RS calculation, linear conversion was then performed (scores ranging from 0 to 100), according to the following calculation:


$$Escore_{decadadimensão}=\left[\left(EB-1\right)/2\right]\times 100$$

For the variable “frequency of care”, a scoring scale ranging from 0 to 3 was assigned according to the regularity indicated by the participant. The response “never” received a score of 0; “monthly” received a score of 1; “weekly” received a score of 2; and “daily” received a score of 3.

Data collection was conducted through face-to-face interviews carried out by a trained team composed of nurses with mental health training who had previously been instructed using a standardized procedures manual to ensure uniformity in the approach and application of the instruments. Initially, managers of the authorized BHUs were contacted to organize the logistics of data collection and facilitate access to professionals. The research team visited the units on dates and shifts previously agreed upon with local management. PHC nurses were approached in their workplace and received explanations regarding the study objectives, participation criteria, confidentiality, and interview duration. Those who agreed to participate were interviewed in a private room to ensure privacy and comfort. Interviews were conducted in a standardized manner, strictly following the established protocol, and the collected data were entered and stored directly in the REDCap platform.

### Data Analysis

**Figure 1:**
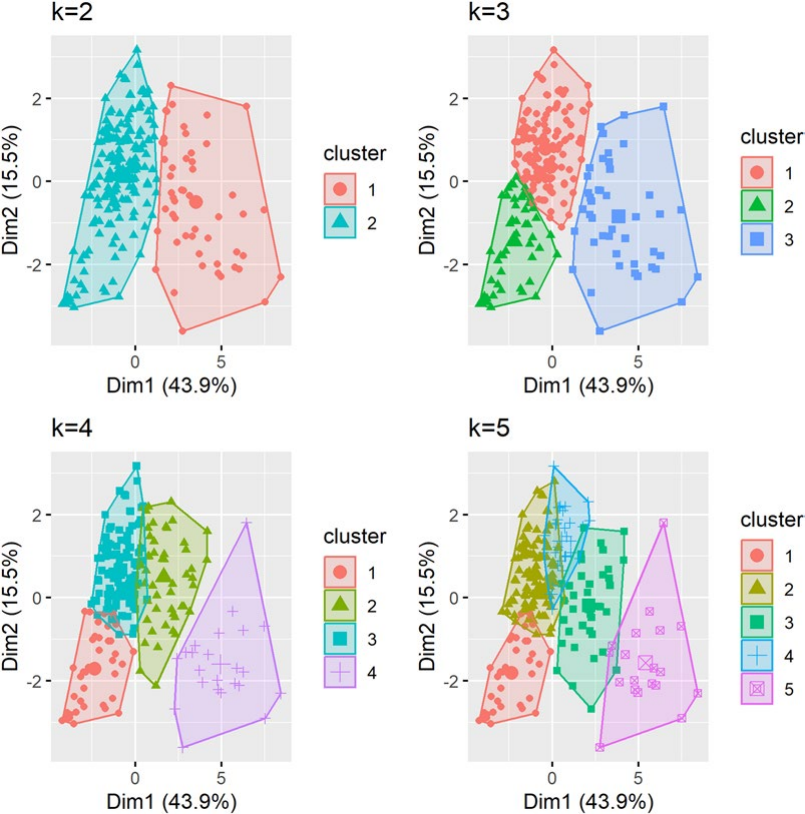
Determination of clusters for analysis – São Paulo, SP, Brazil, 2023.

Data were analyzed using descriptive statistics. To compare the variables of interest according to the calculated scores, the nonparametric Mann-Whitney or Kruskal-Wallis tests were used, depending on the number of categories of each independent variable. To identify distinct nurse profiles with the highest scores, hierarchical cluster analysis was performed based on the following variables: sex; age group; marital status; training in mental health; training in alcohol, drugs, and health; training in substance dependence; and training in mental health. The variables included in the cluster analysis were selected based on their theoretical and empirical relevance for characterizing professional profiles, representing the professional competency cycle: knowing one’s role (professional responsibility), having the means to perform it (feasibility), knowing how to perform it (preparedness), and ultimately performing it (frequency). This selection was based on data from the literature, including variables identified as associated with the studied phenomenon^(6,8,16,17)^. The validity and stability of the cluster solution were ensured through a convergence-of-methods approach. First, the parameter nstart = 25 was used in the k-means algorithm to ensure that the final solution did not represent a local maximum. The cluster structure was validated using the Silhouette index, which showed a mean coefficient of 0.38, confirming a cluster solution consistent with the literature on behavioral and health studies, in which values above 0.3 are considered acceptable to justify the existence of groups. Although cluster 1 showed greater cohesion (mean width of 0.46) and cluster 2 greater heterogeneity (0.10), Principal Component Analysis demonstrated complete separation of the centroids without overlap for k = 2, ensuring the stability of the binary partition (Figure 1). Finally, face validity was demonstrated through visual comparison of the means, in which the groups presented relevant differences across all 12 analyzed scores (Table 1).

**Table 1:** Distribution of participant responses to interventions by component and dimension in Primary Health Care – São Paulo, SP, Brazil, 2023.

Interventions/component	Dimension	Yes (%)	No (%)
Component I interventions	Nurses’ role in Primary Health Care	90.4%	9.6%
	Feasibility of implementation at the Basic Health Unit	91.3%	8.7%
	Perception of professional skills	58.9%	41.1%
Component II interventions	Nurses’ role in Primary Health Care	84.1%	15.9%
	Feasibility of implementation at the Basic Health Unit	86.7%	13.3%
	Perception of professional skills	48.6%	51.4%
Component III interventions	Nurses’ role in Primary Health Care	90.9%	9.1%
	Feasibility of implementation at the Basic Health Unit	92.6%	7.4%
	Perception of professional skills	58.5%	41.5%

To provide greater reliability for interpreting the results, the internal consistency of the components comprising Section III of the instrument, related to frequency, professional responsibility, and preparedness, was assessed. Cronbach’s alpha coefficient was calculated for this purpose. All analyses were conducted using R software and the “cluster” and “factoextra” packages. To identify the association between the variables of interest and the clusters, a log-binomial regression model was used to estimate prevalence ratios^(18)^. A significance level of 5% was adopted for all analyses.

### Ethical Considerations

O The study was approved by the Research Ethics Committee of the host institution under Opinion 5,592,768. All participants who agreed to participate in the study received and signed the Informed Consent Form. Risks and benefits were explained, and confidentiality was guaranteed to all participants. Data were stored in an encrypted database accessible only to the research team.

## RESULTS

### Sample Sociodemographic and Educational Characteristics

The sample consisted predominantly of female participants (88.2%), with a mean age of 40.9 years (SD = 7.75), married (55.65%), graduated from private institutions (82.43%), and with a mean time since graduation of 12 years (SD = 5.25). Participants had worked in PHC between 5 and 10 years. Regarding graduate education, 95.4% had completed *lato sensu* graduate studies. Concerning the preparation received during nursing education to work in mental health within PHC, 75.2% of respondents reported not having received such preparation, while 60.6% also reported not having received preparation to work with individuals with needs related to substance use. In addition, 80% of respondents considered that undergraduate education had not prepared them for this activity, and 90% identified insufficient coverage of this content during undergraduate training. The main form of training reported by those who stated they had received some preparation during undergraduate education was through lectures and extracurricular activities. When asked about continuing education and in-service training, 75.6% and 72.2% of respondents reported not having participated in any training activities related to substance use and mental health, respectively, within the previous five years.

### Professional Responsibility, Feasibility of Implementation, and Perceived Preparedness to Perform Interventions in Primary Health Care

In the comparative analysis of the components, the highest percentage of professional responsibility was observed in component III (Educational and managerial interventions) (90.9%). The perception of feasibility of implementing the intervention at the BHU was also highest in this component (92.6%). Regarding perceived professional preparedness, component I (Universal practice interventions), which includes interventions that interface with other PHC care lines, presented a slight advantage (58.9%) compared with the other components. In contrast, the lowest percentages across all three dimensions were found in component II (Specific mental health and psychiatric interventions): 84.1% (PHC nurses’ role), 86.7% (feasibility of implementation in PHC), and 48.6% (perception of preparedness).

### Perceived Frequency of Care for Mental Health Conditions

When asked about the frequency of care for mental health conditions, 48.4% of professionals reported providing daily care, 28.4% weekly care, and 20.9% monthly care. Analysis of responses regarding the four investigated conditions revealed a predominance of daily care for anxiety (71.1%), depression (61.5%), and diffuse and nonspecific psychological distress (54.8%). Schizophrenia cases, however, were mostly managed on a monthly basis (57.7%).

### Measure Reliability

In the analysis of the internal consistency of the components of Section III of the instrument, Cronbach’s alpha coefficients indicated good internal consistency of the intervention sets (component I = 0.79, component II = 0.90, and component III = 0.77), suggesting the reliability of the listed components.

### Cluster Analysis

Cluster analysis resulted in two profiles. Cluster 1 grouped professionals with higher scores across all components (Table 2).

**Table 2:** Distribution of average scores obtained by participants in components I, II, and III according to the assessed dimensions (nurses’ role in Primary Health Care; possibility of implementation in Primary Health Care; and perception of training and frequency of execution) and the identified clusters (n = 239) – São Paulo, SP, Brazil, 2023.

Variable	Cluster	
	1 (n = 187) Mean (SD)	2 (n = 52) Mean (SD)
Role regarding component I	96.2* (6.7)	77.1 (18.7)
Role regarding component II	92.3 (10.4)	60.2 (22.2)
Role regarding component III	98.2 (6.7)	69.9 (29)
Possibility of implementation regarding component I	97.0 (5.9)	80.6 (16.2)
Possibility of implementation regarding component II	93.5 (8.1)	68.2 (20.2)
Possibility of implementation regarding component III	98.0 (7.3)	77.6 (26.4)
Feeling trained regarding component I	78.6 (13.4)	54.0 (15.1)
Feeling trained regarding component II	71.2 (16.9)	43.8 (19.7)
Feeling trained regarding component III	80.7 (18.9)	51.3 (22.8)
Frequency of performance regarding component I	64.6 (15.6)	51.0 (9.9)
Frequency of performance regarding component II	49.8 (19.9)	31.5 (13.9)
Frequency of performance regarding component III	54.6 (21.5)	40.6 (18.3)

SD – standard deviation;

*Maximum possible score 100 points.

The log-binomial regression analysis indicated a statistically significant association between training in substance dependence and cluster distribution, suggesting that professionals with training in this area were more prevalent in cluster 1. Regarding age group, professionals up to 59 years old showed a lower prevalence in cluster 2, indicating a predominance of younger individuals in cluster 1. On the other hand, the variables sex, marital status, having received training in mental health or alcohol and other drugs during undergraduate education, and having received mental health training did not show statistically significant associations with the identified clusters. Overall, cluster 1 was composed of younger nurses with greater specific qualification for caring for individuals who use psychoactive substances (Table 3).

**Table 3:** Distribution of variables that showed a significant association with the identified clusters (n = 239) – São Paulo, SP, Brazil, 2023.

Variable	Cluster		PR* (95% CI) (cluster = 1)	p-value	PR (95% CI) (cluster = 2)	p-value
	1 (n%)	2 (n%)				
Age group						
Up to 44 (n = 160)	126 (78.7%)	34 (21.2%)	2.363 (0.348; 16.051)	0.544	**0.319 (0.115; 0.885)**	**0.024**
Between 45 and 59 (n = 65)	52 (80%)	13 (20%)	2.4 (0.352; 16.356)	0.533	**0.3 (0.098; 0.919)**	**0.032**
60 or older (n = 3)**	1 (33.3%)	2 (66.7%)	ref.		ref.	
Training in chemical dependency						
No (n = 180)	135 (75%)	45 (25%)	ref.		ref.	
Yes (n = 58)	51 (87.9%)	7 (12.1%)	**1.172 (1.032; 1.332)**	**0.014**	0.483 (0.231; 1.011)	**0.054**

*PR – Prevalence Ratio estimated by a log-binomial regression model; 95% CI – 95% Confidence Interval;

**Categories with reduced absolute frequency (n < 5) were retained to preserve the integrity of the original sample, although the resulting confidence intervals should be interpreted with caution due to lower statistical precision.

## DISCUSSION

This study aimed to identify the variables associated with nurses’ perceptions regarding the recognition of mental health demands and the implementation of interventions in the PHC context. It was found that component II (Specific mental health interventions) presented the lowest level of self-perceived preparedness among professionals (48.6%). Even so, most participants considered these interventions feasible (86.7%) and compatible with their professional responsibilities (84.1%). Analysis of the perceived frequency of care for mental health conditions revealed a predominance of CMDs, such as anxiety, depression, and psychological distress, which were frequently reported as part of nurses’ daily care activities, indicating a high demand for and strong presence of these conditions within the PHC care routine^(2,19)^. In contrast, persistent and severe mental disorders, such as schizophrenia, were reported less frequently, with monthly care provision, which may be related to the greater prevalence of CMDs in the population compared with SMDs (0.3% to 1%)^(20)^.

In this context, the low prevalence of these conditions in PHC may also be related, among other factors, to the recurrent practice of direct referrals to the RAPS^(16,21)^, especially to Psychosocial Care Centers, a phenomenon that may contribute to the low prevalence of this population seeking healthcare within PHC. This phenomenon may discourage the search for healthcare within PHC, despite PHC being considered a strategic setting for the care of individuals with SMDs, especially due to clinical monitoring, support for treatment adherence, and coordination with the healthcare network, which favor continuity of care and reduce hospitalizations^(22,23)^.

Considering the absence of reliable instruments encompassing the investigated variables (professional responsibility, feasibility, and frequency), it was necessary to verify the internal consistency of the components of Section III of the questionnaire as an initial step to ensure the reliability of the obtained data. Satisfactory levels of reliability were observed, as measured by Cronbach’s alpha coefficient^(24)^, indicating good coherence among the items composing each dimension and demonstrating that the instrument showed adequate stability for measuring the assessed constructs. Although the findings indicate satisfactory levels of internal consistency, evidenced by Cronbach’s alpha coefficients, it is important to recognize that these results do not exhaust the psychometric assessment of the instrument. The performed analysis addresses only one dimension of validity and reliability and does not include, for example, evidence of construct validity (such as factor analysis) or external validity. Therefore, although the indicators point to a coherent internal structure, the instrument should still be understood as being in an initial stage of development and having an exploratory nature.

Cluster analysis revealed the existence of distinct profiles among PHC nurses. Although cluster 1 concentrated most of the sample (78%), this distribution does not indicate low discrimination, but rather reflects the epidemiological profile of the studied healthcare network. The analysis accurately identified a majority group characterized by “standard engagement” and, more importantly, isolated a minority subgroup represented by cluster 2. The subgroup identified in cluster 2 (22%) presented lower scores, especially in component II. However, its interpretation requires caution. The reduced silhouette index for this cluster (0.10) indicates low internal cohesion and weak separation from cluster 1, suggesting that the individuals allocated to this group do not form a clearly delimited set. Therefore, this cluster may partially reflect the grouping of cases that did not adequately fit the predominant pattern observed in cluster 1 rather than a homogeneous group with well-defined characteristics.

Despite the fact that data analysis revealed that cluster 2 does not constitute a clearly defined group due to its low internal cohesion, its results indicate the existence of a subgroup of professionals with greater perceived weaknesses, especially in central dimensions of practice. This finding suggests the need to prioritize structured educational strategies targeted at this group of nurses. Thus, rather than supporting rigid categories, the findings contribute to guiding educational actions aimed at strengthening essential competencies, particularly among those who present greater distance from the predominant perceived pattern.

On the other hand, although cluster 1 demonstrated higher perceptions of feasibility, professional responsibility, and frequency of practices, it also showed limited levels of perceived preparedness in component II (49.8%). This finding suggests a possible misalignment between perceived confidence and technical mastery, which may represent the risk of “illusion of competence”^(10,11,12)^.

In this sense, the two clusters are not merely a division of scores, but rather evidence that weakness in one dimension ultimately affects nurses’ overall performance, corroborating the literature demonstrating that more favorable attitudes, greater technical mastery, and higher levels of self-efficacy tend to promote engagement in psychosocial practices and the incorporation of mental health interventions into PHC^(25,26,27)^. When articulated with institutional support, these elements may explain the greater propensity of professionals in this group to perform specific interventions and adopt broader care strategies, in accordance with findings from recent studies.

Among the assessed components, component III (Educational and managerial interventions) was the most consolidated in nurses’ perceptions of practice. In contrast, component II (Specific mental health and psychiatric interventions) stood out as the most challenging for implementation in PHC. The pattern identified in cluster 1 is consistent with the literature showing that nurses who perceive themselves as better prepared, more competent, and more aware that mental health is part of their professional responsibilities tend to engage more actively in psychosocial care actions within PHC. Studies on mental health practices in PHC indicate that feelings of unpreparedness, fear, and lack of specific qualifications function as important barriers to offering interventions, whereas greater training and organizational support increase team problem-solving capacity^(17,28)^.

The coherence between the high scores observed in components I and III, which indicate recognition and perceived feasibility of mental health interventions (with means above 90), and the low level of preparedness in component II (48.6%) may be explained by barriers identified in the literature. Insufficient mental health education during undergraduate nursing training, work overload, the absence of in-service training, and the lack of curricular integration between mental health and PHC during professional education^(29,30,31)^ compromise practical preparation for this component. In addition, a recent analysis of mental health nursing consultations revealed gaps in management competencies, highlighting the need for specialized training to ensure qualified practice^(32)^.

The mean scores for component II, which encompasses perceptions regarding more specific mental health interventions, such as therapeutic relationships, development of Individual Therapeutic Projects, therapeutic groups, use of scales, and psychosocial rehabilitation practices, were consistently lower than those of components I and III in both clusters. This result suggests that, in participants’ perceptions, organizational and educational actions are more easily implemented, whereas interventions requiring complex clinical and relational competencies demand additional training involving specific knowledge and advanced relational skills^(8,29)^, which are not always included in the education of generalist nurses^(17,28,29,30,31)^. These findings reinforce the need to strengthen mental health education in undergraduate nursing programs through the expansion of practical experiences, inclusion of active learning methodologies, and integration between education and healthcare services. They also imply the development of continuing education programs with practical components and clinical supervision, as well as the implementation of institutional policies prioritizing routines and protocols focused on psychosocial care for the population.

The combination of deficient education and lack of continuing professional development contributes to the maintenance of care gaps, negatively impacting the quality of care. Even when mental health demands are identified, many professionals demonstrate difficulties in applying the basic knowledge required for mental health interventions in PHC^(30,33)^. Added to this is the still common perception among professionals that such practices belong exclusively to specialized RAPS services, which tends to restrict their adoption within PHC^(34,35)^, contributing to excessive referrals^(16,21)^ and resulting in reduced demand for healthcare in these settings among individuals with persistent SMDs.

Among the investigated variables, a significant association was observed between training in substance dependence and belonging to cluster 1, suggesting that nurses who had participated in training activities related to substance dependence demonstrated greater confidence and ability to work in psychosocial practices. A systematic review^(36)^ showed that training focused on substance use disorders significantly improves nurses’ technical knowledge, professional attitudes, and self-confidence. This finding corroborates studies indicating training as a critical factor for improving mental healthcare in PHC, especially when directed toward highly stigmatized conditions such as problematic psychoactive substance use^(34,35,37,38)^.

The presence of younger professionals in cluster 1 suggests that these professionals may be more available to perform mental health interventions within their professional practices and may also be more familiar with current psychosocial care guidelines^(39)^. Due to their shorter time since graduation, younger nurses tend to have access to more updated curricula, which favors the adoption of integrated and evidence-based practices in PHC. In addition, the literature demonstrates advances in discussions regarding nursing curriculum guidelines, emphasizing the need to enhance the value of mental health content^(37,38,39)^.

The results of this study reiterate the urgent need for continuing education strategies encompassing not only technical improvement, but also the specificities of professional profiles. Recent evidence highlights that effective educational processes should include participatory methodologies, such as clinical workshops, realistic simulations, case discussions, matrix supervision, and interprofessional education, promoting integration between theory and practice and critical reflection on care. Regarding relational competencies, emphasis is placed on skills such as therapeutic communication, development of qualified listening, empathy, bond-building, negotiation of care plans, shared decision-making, and the ability to act in crisis situations with a welcoming and non-coercive approach. These competencies are considered central to nurses’ performance in the early identification of mental health demands, establishment of trust-based relationships, and implementation of evidence-based clinical mental health interventions^(7)^.

Based on the results and their practical and policy implications for PHC and mental health nursing education, there is a clear need to expand and formalize mental health nursing consultations within PHC, recognizing their potential to increase access, strengthen therapeutic bonds, and improve psychosocial care^(40)^. The systematic integration of mental health into the routine of PHC as a central axis of comprehensive care requires the reorganization of work processes and effective coordination among the different points of the healthcare network^(25)^.

At the same time, there is an urgent need to invest in continuing education programs that include practical, relational, and clinical supervision components, which are fundamental for the development and maintenance of the competencies required for nurses’ practice in this field^(41)^. Such measures should be accompanied by formal recognition of nursing as a key actor in community mental health, especially in low- and middle-income countries where there is a shortage of specialists^(42,43)^. Furthermore, institutional recognition of mental health nursing as a legitimate and strategic field of practice should be emphasized, involving adequate working conditions, organizational support, and professional visibility. Finally, the results point to the need for educational and regulatory policies that strengthen mental health education during undergraduate training and standardize essential competencies for professional practice, thereby contributing to sustainably improving the PHC response to psychosocial demands.

### Study Limitations

The results of this study provide relevant findings regarding the role of nurses in PHC and highlight persistent gaps in their preparedness to perform specific mental health interventions. In addition, the study provides evidence regarding professionals’ perceptions, as well as the perceived barriers and potentialities that structure their clinical practice. Thus, it contributes to advancing knowledge about the factors influencing the identification of mental health demands and the implementation of interventions within PHC, a field that remains underexplored in the national literature. The study also provides evidence regarding perceptions, barriers, and potentialities of professional mental health practice in this context, identifying groups of professionals with a greater propensity to engage in mental health actions within PHC. However, some limitations should be considered when extrapolating the results. Although the data collection instrument was developed to identify and describe mental health nursing interventions in PHC, it was not designed to measure a specific latent construct. The instrument was mainly based on institutional regulations, evidence from the literature, and expert content assessment, seeking to ensure the relevance of the interventions to the scope of nursing practice. It should be recognized as a limitation that more robust psychometric analyses aimed at assessing construct validity and, consequently, formal theoretical grounding were not conducted. Future studies may further investigate the psychometric properties of the proposed instrument, including factor analyses and other validity approaches, in order to increase the methodological robustness of the tool. Furthermore, no data were collected regarding the practice and frequency of care for harmful psychoactive substance use conditions, which restricts the scope of the analysis. As this was a cross-sectional study, it is not possible to establish causal relationships among the studied variables. The decision to use a three-point scale instrument to facilitate faster completion may have resulted in lower sensitivity of the scale to capture more subtle nuances and variations in participants’ perceptions, potentially reducing discrimination between intermediate levels of agreement or preparedness. In addition, the low representativeness of certain demographic categories, such as professionals aged 60 years or older (n = 3), should be highlighted, as this resulted in wide 95% Confidence Intervals and low statistical power in the log-binomial regression for this specific variable. Nevertheless, these strata were retained to preserve the reliability of the collected sample. Therefore, findings regarding age group should be interpreted as indicative trends requiring confirmation in studies with larger samples. Partial adherence of the BHUs may also introduce selection bias, since participating units may demonstrate greater interest, organization, or sensitivity toward mental health issues compared with units that did not participate. Moreover, another limitation of the study refers to the possible social desirability bias, since responses were self-reported by participants. Under these conditions, nurses may have tended to report practices considered more appropriate or socially expected within their professional context. Although response anonymity was ensured, the influence of this type of bias on the results cannot be completely ruled out. These factors may compromise the accuracy of the collected information. Although the sample is representative of different regions of an important Brazilian city, generalization of the findings to other contexts should be approached with caution. Finally, as a central limitation, it should be emphasized that the findings are based on nurses’ self-perceptions regarding feasibility, professional responsibility, and preparedness to perform interventions, which does not allow direct inferences about their actual implementation in clinical practice. This possible dissociation between perception and actual practice may overestimate or underestimate professional performance within the PHC context. Therefore, caution is recommended when interpreting the results, as well as the development of future studies including observational measures or objective indicators of clinical practice.

## CONCLUSION

Strengthening nurses’ psychosocial competencies is strategic for sustainably improving the PHC response to mental health demands. The results indicate the need to invest in specific education, continuing professional development, and institutional support, especially directed toward the different professional profiles identified.

## Data Availability

The entire dataset supporting the results of this study has been published within the article itself.
