# Supplementary material for: Mental health interventions performed by nurses in Primary Health Care: analysis according to professional profile
Source: Rev Esc Enferm USP. 2026 Jul 20;60:e20250463. doi: 10.1590/1980-220X-REEUSP-2025-0463en (PMC13384509; doi:10.1590/1980-220X-REEUSP-2025-0463en)
Supplement: Translation

# Translation: Intervenções de saúde mental realizadas por enfermeiros na Atenção Primária à Saúde: análise segundo o perfil profissional*

**DOI:** 10.1590/1980-220X-REEUSP-2025-0463pt

**Authors:** Rafaela Sales Medeiros, Divane de Vargas, Jaqueline Lemos de Oliveira

**Affiliations:** 1 Universidade de São Paulo, Escola de Enfermagem, São Paulo, SP, Brasil.

**Keywords:** Enfermeiras e Enfermeiros, Assistência à Saúde Mental, Atenção Primária à Saúde

## Abstract

**Objetivo:**

Identificar as variáveis relacionadas à percepção de enfermeiros em reconhecer demandas de saúde mental e implementar intervenções no contexto da Atenção Primária à Saúde.

**Método:**

Estudo transversal, realizado com 239 enfermeiros da Atenção Primária à Saúde. As intervenções identificadas foram agrupadas em três componentes: intervenções universais; específicas de saúde mental e psiquiatria; e educativas e gerenciais. Os participantes avaliaram viabilidade de execução, relação com seu escopo de atuação e autopercepção de capacitação para executá-las, respondendo a um instrumento estruturado, submetido à avaliação de conteúdo por especialistas e teste piloto, que apresentou consistência interna satisfatória (α = 0,77–0,90). A análise de *cluster* foi utilizada para identificar perfis profissionais distintos quanto à percepção de atribuição, viabilidade, capacitação e frequência de realização das intervenções. Em seguida, aplicou-se regressão log-binomial para analisar a associação entre as variáveis de interesse e os *clusters* identificados.

**Resultados:**

Entre os participantes, 48,4% relataram atendimentos diários a demandas em saúde mental, com destaque para ansiedade (71,1%). A análise dos *clusters* indicou que profissionais mais jovens e com formação específica em dependência química apresentaram maiores pontuações em todas os componentes investigados. O componente referente às intervenções específicas em saúde mental teve as menores pontuações e percepção de capacitação.

**Conclusão:**

É necessário ampliar investimentos na formação, no treinamento e na capacitação dos enfermeiros para a realização de intervenções em saúde mental na Atenção Primária à Saúde.

## INTRODUÇÃO

Os transtornos mentais constituem um conjunto heterogêneo de condições que variam de acordo com a intensidade, o curso e o impacto funcional. A literatura tem diferenciado, de forma cada vez mais precisa, os transtornos mentais comuns (TMC) dos transtornos mentais graves (TMGs). Os TMCs, como depressão leve a moderada, ansiedade e queixas somatoformes, apresentam alta prevalência na Atenção Primária à Saúde (APS) e estão associados a sofrimento psíquico significativo, embora geralmente não impliquem prejuízo severo da autonomia ou ruptura dos vínculos sociais. Em contraste, os TMGs, que incluem esquizofrenia, transtornos psicóticos persistentes, transtorno bipolar e quadros severos de uso/abuso de substâncias, demandam acompanhamento contínuo, maior complexidade assistencial e articulação estreita com os serviços da Rede de Atenção Psicossocial (RAPS). Estima-se que oito em cada dez pessoas com TMGs não recebem tratamento^(1)^. A combinação de alta prevalência e baixa identificação reforça a urgência de respostas estruturadas no sistema de saúde.

Diante do exposto, a integração da saúde mental à APS tem sido apontada como estratégia-chave para a redução desses problemas nas comunidades^(2)^. Por sua capilaridade e proximidade com os territórios, a APS é capaz de ampliar o acesso, promover a detecção precoce e oferecer intervenções oportunas para mitigar a invisibilidade e o não tratamento dos problemas de saúde mental. Apesar disso, a integração dos cuidados à saúde mental na APS ainda enfrenta diversos desafios, particularmente em contextos com escassez de recursos. A ausência de profissionais capacitados, o financiamento insuficiente e a fragilidade da educação permanente são barreiras recorrentes. Soma-se a isso a persistente falta de conscientização e de formação adequada entre médicos, enfermeiros e agentes comunitários, o que contribui para o estigma e a discriminação em torno dos transtornos mentais^(3,4)^.

As ações realizadas por não especialistas podem ampliar a capacidade dos serviços de saúde mental na linha de frente e melhorar significativamente o cuidado. Nesse contexto, especialmente em países de baixa e média renda, como o Brasil, onde o acesso a profissionais especializados é crítico, os enfermeiros constituem-se como recursos importantes para o enfrentamento desses problemas. Isso porque os enfermeiros da APS frequentemente constituem o primeiro ponto de contato dos usuários com o sistema de saúde. Para além dos cuidados físicos, desempenham um papel central no cuidado em saúde mental, especialmente no estabelecimento de vínculo, na escuta qualificada e na comunicação terapêutica. Essas dimensões do cuidado em saúde mental permitem que os enfermeiros desenvolvam intervenções para o manejo de crises e a identificação precoce de sinais de TMC e TMG. Dessa forma, eles contribuem não só para a melhoria da capacidade do serviço diante de quadros relacionados à saúde mental, como também para a articulação com a RAPS^(5,6)^.

Entretanto, apesar do potencial desses profissionais no enfrentamento da problemática, os mesmos ainda encontram dificuldades em aumentar seu escopo de ações em saúde mental na APS^(7)^. A escassez de capacitações profissionais, a pouca atenção dada à questão durante a formação^(8,9)^ e o estigma diante da doença mental^(3)^ contribuem para a baixa identificação e utilização das intervenções de enfermagem em saúde mental como uma prática inerente ao cenário da APS^(7)^.

Apesar de bem conhecidas as barreiras encontradas para a implementação de ações de saúde mental na APS pelos enfermeiros^(7,8,9)^, frequentemente, tais achados são investigados a partir da percepção dos próprios trabalhadores. Entretanto, a literatura aponta que a percepção de competência nem sempre corresponde ao desempenho efetivo na prática clínica. A teoria da autoeficácia sustenta que a crença do indivíduo em sua capacidade de executar determinadas ações influencia seu comportamento e tomada de decisão, porém tal percepção pode não refletir necessariamente o nível real de competência ou a qualidade da execução das práticas assistenciais^(10,11)^. Nesse sentido, estudos na área da educação e das profissões da saúde têm discutido a existência de um *gap* entre a autopercepção de habilidades e o desempenho observado, denominado risco de “ilusão de competência”^(10,11,12)^. Isso reforça a importância de abordagens baseadas em competências que considerem não apenas a confiança do profissional, mas também a mobilização efetiva de conhecimentos, habilidades e atitudes na prática clínica.

Não foram identificados estudos publicados que permitam identificar perfis distintos de enfermeiros segundo características sociodemográficas, formativas e de capacitação que evidenciem grupos com maior ou menor propensão à inserção de ações de saúde mental em sua prática cotidiana na APS. Assim, investigações que possibilitem a identificação de possíveis diferenciais entre grupos específicos avançam em relação às pesquisas anteriores, que em geral descrevem práticas de forma agregada, sem discriminar padrões internos de heterogeneidade profissional. Uma análise considerando esses aspectos possibilita direcionar estratégias formativas e de gestão mais precisas, alinhadas às necessidades específicas do perfil profissional, fortalecendo a institucionalização do cuidado de enfermagem em saúde mental na prática da APS.

Diante desse cenário, compreender os fatores que influenciam a atuação do enfermeiro, considerando aspectos formativos, institucionais e subjetivos que determinam sua capacidade de identificar e implementar tais intervenções, torna-se importante. Buscando responder à pergunta “Quais variáveis (sociodemográficas, formativas e ou institucionais) estão associadas à maior identificação e realização de intervenções em saúde mental por enfermeiros que atuam na APS?”, este estudo teve como objetivo identificar as variáveis relacionadas à percepção desses profissionais em reconhecer demandas de saúde mental e implementar intervenções no contexto da APS.

## MÉTODO

### Delineamento do Estudo

Trata-se de estudo transversal baseado na ferramenta *STrengthening the Reporting of OBservational studies in Epidemiology*.

### Local e Período da Coleta

Segundo a Secretaria Municipal de Saúde, à época da coleta, o município contava com 468 Unidades Básicas de Saúde (UBSs). Dessas, 81 eram híbridas, com atendimento associado a especialidades (ambulatórios médicos de especialidades), e foram excluídas do estudo, resultando em um total de 387 UBSs tradicionais, as quais foram incluídas no estudo. Todos os gerentes das potenciais unidades participantes foram contatados individualmente por e-mail e por telefone para esclarecimentos sobre os objetivos do estudo e para solicitar autorização para a realização da coleta. Ao final, 66 (17 %) UBSs aceitaram participar e compuseram o cenário da coleta de dados, que ocorreu entre março e agosto de 2023.

### Amostra

A amostra foi composta por enfermeiros atuantes nas unidades participantes do estudo. Os critérios de inclusão foram: a) possuir vínculo empregatício na instituição por, no mínimo, seis meses, tempo considerado suficiente para assegurar a experiência operacional no território e a exposição às rotinas de atenção à saúde mental; e b) ter realizado pelo menos um atendimento em saúde mental, entendido como qualquer consulta, acolhimento ou encaminhamento relacionado às queixas psíquicas, aos sinais de sofrimento mental, ao uso problemático de substâncias ou ao seguimento de pessoas com TMCs ou TMGs. Considerando o número total de enfermeiros vinculados à Secretaria Municipal de Saúde e lotados nas UBSs envolvidas no momento da coleta (N = 403), estabeleceram-se, para o cálculo amostral, uma margem de erro de 5%, uma estimativa de recusas e perdas de 5%, a inclusão de 20% para controle de potenciais fatores de confusão e um nível de confiança de 95%, resultando em uma amostra mínima estimada de 207 participantes. Ao final da coleta, 239 enfermeiros atenderam aos critérios e todos aceitaram participar do estudo. Para assegurar a representatividade territorial e a precisão das estimativas, adotou-se amostragem estratificada, proporcional ao número de enfermeiros por região administrativa. A distribuição final dos participantes incluiu oito enfermeiros da região Central, 69 da região Sul, 19 da região Oeste, 38 da região Norte e 73 da região Leste. Essa estratégia garantiu a inclusão equilibrada dos diferentes territórios e refletiu a heterogeneidade organizacional e demográfica da rede de APS do município de São Paulo.

### Coleta de Dados

Para coleta de dados, utilizou-se um questionário criado pelos autores, contendo 43 questões distribuídas em três seções: Seção I – envolvendo 16 variáveis que investigavam questões relacionadas ao perfil, à formação acadêmica e à formação específica em saúde mental, além das variáveis sociodemográficas como sexo, idade, estado civil, caracterização da instituição formadora (pública, privada e outras), tempo de formação (em meses/anos), preparo para atuação em saúde mental e/ou dependência química durante a graduação (buscou identificar se os conteúdos foram predominantemente ofertados por meio de aulas expositivas, palestras, seminários, discussões orientadas pelo docente ou atividades extracurriculares); Seção II – perfil da formação específica em saúde mental e atendimento em dependência química, envolvendo sete variáveis que investigam se o profissional tem pós-graduação e recebeu capacitação em saúde mental ou em dependência química após a graduação, e a frequência de atendimento dos principais quadros de saúde mental (depressão, ansiedade, esquizofrenia e sintomas somáticos de sofrimento psíquico); Seção III – composta por 20 intervenções de enfermagem em saúde mental na APS, descritas em normativas e documentos técnicos nacionais e municipais, incluindo os Cadernos de Atenção Básica do Ministério da Saúde, as Diretrizes de Atenção Básica da Secretaria Municipal de Saúde de São Paulo^(13,14)^ e a Resolução COFEN nº 599/2018^(15)^, que dispõem sobre a atuação da enfermagem em saúde mental. Adicionalmente, selecionaram-se também intervenções descritas em uma revisão de evidências sobre práticas de enfermagem em saúde mental na APS^(7)^. Essas intervenções foram agrupadas, conforme as Diretrizes de Atenção Básica da Secretaria Municipal de Saúde de São Paulo^(14)^ , em três componentes: Componente I – Intervenções de prática universais, abrangendo intervenções que possuem interface com outras linhas de cuidado da APS; Componente II – Intervenções de prática específica de saúde mental e psiquiatria; e Componente III – Intervenções baseadas em atividades educativas e gerenciais, relacionadas ao preparo da equipe de enfermagem para atuação diante das situações de saúde mental e registro (Quadro 1).

**Quadro 1:** Distribuição das intervenções de acordo com a classificação nos três componentes de intervenções – São Paulo, SP, Brasil, 2023.

Intervenções do componente I	Intervenções do componente II	Intervenções do componente III
1. Acolhimento	3. Aconselhamento	16. Educação em saúde
2. Triagem	5. Relacionamento terapêutico	17. Educação permanente
4. Consulta de enfermagem	7. Segurança do paciente relacionada ao uso de psicotrópicos)	20. Registro de enfermagem
6. Acompanhamento pré-natal	8. Encaminhamentos/apoio matricial	
10. Atendimento domiciliar	9. Apoio social	
15. Práticas integrativas e complementares	11. Apoio familiar	
19. Gerência dos casos	12. Projeto Terapêutico Singular	
	13. Grupos terapêuticos	
	14. Aplicar escalas	
	18. Práticas avançadas em reabilitação psicossocial	

Embora as intervenções tenham sido extraídas de fontes normativas e científicas, que já as reconheciam como práticas válidas e recomendadas no cenário nacional, procedeu-se à avaliação por juízes especialistas com o objetivo de verificar a adequação da alocação dessas intervenções nos três componentes propostos^(14)^. Para isso, cinco juízes participaram do processo, sendo dois com experiência na área de APS e três na área da saúde mental. Os especialistas analisaram a pertinência e a adequação de cada uma das 20 intervenções em relação aos respectivos componentes, considerando também se tais intervenções estavam alinhadas ao escopo das ações desenvolvidas por enfermeiros nos serviços de saúde. Esse procedimento buscou assegurar maior coerência entre as intervenções e os componentes analíticos adotados. Na sequência, antes da coleta definitiva de dados, foi realizado um teste piloto com a aplicação do instrumento a oito enfermeiros pertencentes à população-alvo do estudo. Esse procedimento teve como finalidade avaliar a clareza, a compreensão e a adequação das questões às dimensões propostas. O teste piloto permitiu ainda verificar a consistência da estrutura do questionário, o tempo médio de preenchimento e a compreensão dos itens e das escalas de resposta pelos participantes, possibilitando ajustes pontuais antes da aplicação final do instrumento. Os resultados dessas fases evidenciaram boa compreensão dos itens e adequação da alocação das intervenções nos componentes propostos, não sendo necessárias alterações adicionais antes da aplicação final.

Para cada intervenção listada nos três componentes, os participantes foram questionados quanto à frequência de realização da intervenção na prática cotidiana, se a reconheciam como uma atribuição do enfermeiro de APS e a percepção sobre sua capacitação para executá-la. Para a criação dos nove escores dos componentes I, II e III, estabeleceu-se a pontuação 3 para respostas “sim”, 2 para “não tenho certeza” e 1 para “não” às perguntas “Você considera que essa intervenção é possível ser realizada na UBS?” e “Na sua avaliação, essa é uma atribuição do enfermeiro na APS?”. Para a pergunta “Você se sente capacitado para realizar essa intervenção?”, estabeleceu-se a pontuação 3 para respostas “totalmente”, 2 para “parcialmente” e 1 para “incapaz”. Considerando que a coleta de dados ocorreu durante o horário de trabalho dos enfermeiros, optou-se por uma escala Likert de três pontos, a fim de facilitar a compreensão, reduzir o tempo de resposta e minimizar ambiguidades, além de favorecer uma maior consistência nas respostas. Para a interpretação, os escores de cada pergunta, considerando cada componente, foram calculados de forma a gerar uma pontuação entre 0 e 100, sendo que quanto maior a pontuação, melhor. Considerando as respostas de cada item como uma escala Likert de 3 pontos, o processo se iniciou com o cálculo do escore bruto (EB) do instrumento. Esse EB foi obtido pela média das pontuações dos itens de cada dimensão (Quadro 1).

Esse cálculo foi realizado utilizando a seguinte fórmula:


$$EB=\frac{q1+q2+\cdots +qn}{n}$$

A partir do cálculo do EB, foi realizada a conversão linear (escores de 0 a 100), conforme o seguinte cálculo:


$$Escore_{decadadimensão}=\left[\left(EB-1\right)/2\right]\times 100$$

Para a variável “frequência de atendimento”, foi atribuída uma escala de pontuação de 0 a 3, conforme a regularidade indicada pelo participante. A resposta “nunca” recebeu pontuação 0; “mensalmente” foi pontuada como 1; “semanalmente” foi pontuada como 2; e “diariamente” foi pontuada como 3.

A coleta de dados foi realizada por meio de entrevistas presenciais conduzidas por uma equipe treinada, composta por enfermeiros com formação em saúde mental e previamente capacitados em um manual padronizado de procedimentos, visando assegurar uniformidade na abordagem e na aplicação dos instrumentos. Inicialmente, os gerentes das UBSs autorizadas foram contatados para organizar a logística da coleta e facilitar o acesso aos profissionais. A equipe de pesquisa deslocou-se às unidades em dias e turnos previamente combinados com a gestão local. Os enfermeiros atuantes na APS foram contatados no próprio local de trabalho, onde receberam explicações sobre os objetivos do estudo, os critérios de participação, a confidencialidade e a duração da entrevista. Aqueles que aceitaram participar foram entrevistados em sala reservada, garantindo privacidade e conforto. As entrevistas foram conduzidas de forma padronizada, seguindo rigorosamente o protocolo estabelecido, e os dados coletados foram digitados e armazenados diretamente na plataforma REDCap.

### Análise de Dados

Os dados foram analisados por meio de estatística descritiva. Para comparar as variáveis de interesse quanto às pontuações calculadas, foram utilizados testes não paramétricos de Mann-Whitney ou Kruskall-Wallis, dependendo do número de categorias de cada variável independente. Para identificar perfis distintos de enfermeiros com as maiores pontuações, foi realizada uma análise de *cluster* do tipo hierárquica, com base nas seguintes variáveis: sexo; faixa etária; estado civil; formação em saúde mental; formação em álcool drogas e saúde; capacitação em dependência química; e capacitação em saúde mental. As variáveis incluídas na análise de *cluster* foram selecionadas com base em sua relevância teórica e empírica para a caracterização de perfis profissionais, representando o ciclo de competência profissional: saber qual é a sua função (atribuição), ter meios para executá-la (possibilidade), saber como executá-la (capacitação) e, por fim, executá-la (frequência). Essa escolha baseou-se em dados da literatura, incluindo variáveis apontadas como associadas ao fenômeno estudado^(6,8,16,17)^. A validade e a estabilidade da solução de *cluster* foram garantidas por meio de uma abordagem de convergência de métodos. Primeiramente, utilizou-se o parâmetro *nstart* = 25 no algoritmo *k-means* para assegurar que a solução final não representasse um máximo local. A estrutura dos *clusters* foi validada pelo índice de Silhouette, que apresentou um coeficiente médio de 0,38, confirmando um *cluster* condizente com a literatura de estudos comportamentais e de saúde, na qual valores acima de 0,3 são aceitáveis para justificar a existência de grupos. Embora o *cluster* 1 apresente maior coesão (largura média de 0,46) e o *cluster* 2, maior heterogeneidade (0,10), a Análise de Componentes Principais evidenciou a separação total dos centroides sem sobreposição para k = 2, garantindo a estabilidade da partição binária (Figura 1). Por fim, a validade de face foi demonstrada pela comparação visual das médias, na qual os grupos apresentaram diferenças relevantes em todos os 12 escores analisados (Tabela 1).

**Figura 1:** Determinação dos *clusters* para análise. São Paulo, SP, Brasil, 2023.

**Tabela 1:** Distribuição das respostas dos participantes das intervenções por componente e dimensão na Atenção Primária à Saúde – São Paulo, SP, Brasil, 2023.

Intervenções/componente	Dimensão	Sim (%)	Não (%)
Intervenções do componente I	Atribuição do enfermeiro na Atenção Primária à Saúde	90,4%	9,6%
	Viabilidade de realização na Unidade Básica de Saúde	91,3%	8,7%
	Percepção de capacitação profissional	58,9%	41,1%
Intervenções do componente II	Atribuição do enfermeiro na Atenção Primária à Saúde	84,1%	15,9%
	Viabilidade de realização na Unidade Básica de Saúde	86,7%	13,3%
	Percepção de capacitação profissional	48,6%	51,4%
Intervenções do componente III	Atribuição do enfermeiro na Atenção Primária à Saúde	90,9%	9,1%
	Viabilidade de realização na Unidade Básica de Saúde	92,6%	7,4%
	Percepção de capacitação profissional	58,5%	41,5%

Para conferir maior segurança à interpretação dos resultados, foi verificada a consistência interna dos componentes que compunham a seção III do instrumento, relacionada à frequência, atribuição e capacitação. Para isso, calculou-se o coeficiente alfa de Cronbach. Todas as análises foram conduzidas no *software* R, utilizando os pacotes “*cluster*” e “factoextra”. Para identificar a associação entre as variáveis de interesse e os clusters, foi utilizado um modelo de regressão *log-binomial* para estimação da razão de prevalência^(18)^. Para todas as análises, adotou-se um nível de significância de 5%.

### Considerações Éticas

O estudo foi aprovado pelo Comitê de Ética em Pesquisa da instituição sede, sob Parecer n° 5.592.768. Todos que aceitaram participar da pesquisa receberam uma via e assinaram o Termo de Consentimento Livre e Esclarecido. Explicaram-se os riscos e benefícios, garantindo-se a confidencialidade aos participantes. Os dados foram armazenados em banco criptografado, que é acessível apenas à equipe de pesquisa.

## RESULTADOS

### Caracterização Sociodemográfica e Educacional da Amostra

A amostra é constituída, majoritariamente, por indivíduos do sexo feminino (88,2%), com idade média de 40,9 anos (DP = 7,75), casados (55,65%), graduados em instituições privadas (82,43%) e com tempo médio de formação de 12 anos (DP = 5,25). Eles atuam há entre 5 e 10 anos. Quanto à formação em pós-graduação, 95,4% possuíam pós-graduação *lato sensu*. Quanto ao preparo recebido durante a formação em enfermagem para atuar em saúde mental na APS, 75,2% dos respondentes relataram não o ter recebido, enquanto 60,6% também referiram não ter recebido preparo para atuar com pessoas com necessidades relacionadas ao uso de substâncias. Além disso, 80% dos entrevistados consideraram que a graduação não os preparou para essa atividade, e 90% identificaram a insuficiência desse conteúdo durante a graduação. A principal forma de capacitação referida por aqueles que relataram ter recebido algum preparo durante a formação foi por meio de aulas e atividades extracurriculares. Quando questionados sobre educação continuada e treinamento em serviço, 75,6% e 72,2% dos respondentes informaram não ter participado de qualquer atividade de capacitação em uso de substâncias e saúde mental, respectivamente, nos últimos cinco anos.

### Atribuição, Viabilidade de Execução e Percepção de Capacitação para Executar as Intervenções na Atenção Primária à Saúde

Na análise comparativa dos componentes, o maior percentual de atribuição foi observado no componente III (Intervenções baseadas em atividades educativas e gerenciais) (90,9%). A percepção de viabilidade de realização da intervenção na UBS também foi mais elevada nesse componente (92,6%). Já quando se analisa a percepção sobre a capacitação profissional, o componente I (Intervenções de prática universais), abrangendo intervenções que possuem interface com outras linhas de cuidado da APS, apresentou ligeira vantagem (58,9%) diante dos demais. Por outro lado, os menores percentuais nas três dimensões ocorreram no componente II (Intervenções de prática específica de saúde mental e psiquiatria): 84,17% (atribuição do enfermeiro de APS), 86,7% (viabilidade de realização na APS) e 48,6% (percepção de capacitação).

### Frequência Percebida de Atendimento de Quadros de Saúde Mental

Após serem questionados sobre a frequência de atendimento de quadros de saúde mental, 48,4% dos profissionais informaram realizar atendimentos diários, 28,4% atendimentos semanais e 20,9% atendimentos mensais. Quando se analisam as respostas para o atendimento das quatro condições investigadas, verificou-se predominância de atendimentos diários para ansiedade (71,1%), depressão (61,5%) e sofrimento difuso e inespecífico (54,8%). Já os casos de esquizofrenia foram atendidos, em sua maioria, mensalmente (57,7%).

### Confiabilidade da Medida

Na análise de consistência interna dos componentes da Seção III do instrumento, por meio do cálculo dos coeficientes alfa de Cronbach observou-se que os respectivos coeficientes (componente I = 0,79, componente II = 0,90 e componente III = 0,77) indicaram boa consistência interna dos conjuntos de intervenções, sugerindo a confiabilidade dos componentes listados.

### Análise dos Clusters

A análise por *cluster* resultou em dois perfis. O *cluster* 1 agrupou os profissionais com pontuações maiores em todos os componentes (Tabela 2).

**Tabela 2:** Distribuição das pontuações médias obtidos pelos participantes nos componentes I, II e III de acordo com as dimensões avaliadas (Atribuição do enfermeiro na Atenção Primária à Saúde; Possibilidade de realização na Atenção Primária à Saúde; e Percepção de capacitação e frequência de execução) e os *clusters* identificados (n = 239) – São Paulo, SP, Brasil, 2023.

Variável	*Cluster*	
	1 (n = 187) Média (DP)	2 (n = 52) Média (DP)
Atribuição referente ao componente I	96,2* (6,7)	77,1 (18,7)
Atribuição referente ao componente II	92,3 (10,4)	60,2 (22,2)
Atribuição referente ao componente III	98,2 (6,7)	69,9 (29)
Possibilidade de ser realizada referente ao componente I	97,0 (5,9)	80,6 (16,2)
Possibilidade de ser realizada referente ao componente II	93,5 (8,1)	68,2 (20,2)
Possibilidade de ser realizada referente ao componente III	98,0 (7,3)	77,6 (26,4)
Se sentir capacitado referente ao componente I	78,6 (13,4)	54,0 (15,1)
Se sentir capacitado referente ao componente II	71,2 (16,9)	43,8 (19,7)
Se sentir capacitado referente ao componente III	80,7 (18,9)	51,3 (22,8)
Frequência de realização referente ao componente I	64,6 (15,6)	51,0 (9,9)
Frequência de realização referente ao componente II	49,8 (19,9)	31,5 (13,9)
Frequência de realização referente ao componente III	54,6 (21,5)	40,6 (18,3)

DP – desvio padrão;

*Escore máximo possível 100 pontos.

A análise de regressão *log-binomial* indicou associação estatisticamente significativa entre a capacitação em dependência química e a distribuição nos *clusters*, sugerindo que os profissionais com formação nessa área são mais prevalentes no *cluster* 1. Em relação à faixa etária, profissionais com até 59 anos apresentaram menor prevalência no *cluster* 2, indicando uma predominância de indivíduos mais jovens no *cluster* 1. Por outro lado, as variáveis sexo, estado civil, ter recebido formação em saúde mental ou em álcool e outras drogas durante a graduação, bem como a realização de capacitação em saúde mental, não apresentaram associação estatisticamente significativa com os *clusters* identificados. De modo geral, o *cluster* 1 é composto por enfermeiros mais jovens e com maior qualificação específica para o cuidado de pessoas que fazem uso de substâncias psicoativas (Tabela 3).

**Tabela 3:** Distribuição das variáveis que demostraram associação significativa com os *clusters* identificados (n = 239) – São Paulo, SP, Brasil, 2023.

Variável	*Cluster*		RP* (IC95%) (*cluster* = 1)	Valor de p	RP (IC95%) (*cluster* = 2)	Valor de p
	1 (n%)	2 (n%)				
Faixa etária						
Até 44 (n = 160)	126 (78,7 %)	34 (21,2 %)	2,363 (0,348; 16,051)	0,544	**0,319 (0,115; 0,885)**	**0,024**
Entre 45 e 59 (n = 65)	52 (80 %)	13 (20 %)	2,4 (0,352; 16,356)	0,533	**0,3 (0,098; 0,919)**	**0,032**
60 ou mais (n = 3)**	1 (33,3 %)	2 (66,7 %)	ref.		ref.	
Capacitação em dependência química						
Não (n = 180)	135 (75 %)	45 (25 %)	ref.		ref.	
Sim (n = 58)	51 (87,9 %)	7 (12,1 %)	**1,172 (1,032; 1,332)**	**0,014**	0,483 (0,231; 1,011)	**0,054**

*RP – Razão de Prevalência estimada por um modelo de regressão log-binomial; IC95% – Intervalo de Confiança de 95%;

**Categorias com frequência absoluta reduzida (n < 5) foram mantidas para preservar a integridade da amostra original, embora os intervalos de confiança resultantes devam ser interpretados com cautela devido à menor precisão estatística.

## DISCUSSÃO

O estudo teve como objetivo identificar as variáveis relacionadas à percepção de enfermeiros em reconhecer demandas de saúde mental e implementar intervenções no contexto da APS. Verificou-se que o componente II (Intervenções específicas em saúde mental) apresentou o menor nível de autopercepção de capacitação entre os profissionais (48,6%). Ainda assim, a maior parte deles avaliou essas intervenções como viáveis (86,7%) e compatíveis com suas responsabilidades profissionais (84,1%). A análise da frequência percebida pelos entrevistados dos atendimentos de quadros de saúde mental evidenciou a predominância de TMCs, como ansiedade, depressão e sofrimento psíquico, frequentemente referidos como parte do atendimento diário dos enfermeiros, indicando alta demanda e presença desses agravos na rotina de cuidado em saúde da APS^(2,19)^. Em contrapartida, casos de TMGs e persistentes, como a esquizofrenia, foram relatados como menos frequentes, com atendimentos mensais, o que pode estar relacionado a uma maior prevalência dos TMCs na população em relação aos TMGs (0,3% a 1%)^(20)^.

Ainda nesse contexto, a baixa prevalência desses quadros na APS pode estar relacionada, entre outros fatores, à prática recorrente de encaminhamentos diretos para a RAPS^(16,21)^, especialmente aos Centros de Atenção Psicossocial, fenômeno que pode contribuir para a baixa prevalência dessa população buscando por cuidado de saúde na APS. Esse fenômeno pode desestimular a busca por cuidados de saúde na APS, apesar de ser considerada um espaço estratégico para o atendimento dos TMGs, especialmente pelo monitoramento clínico, apoio à adesão e articulação com a rede, o que favorece a continuidade do cuidado e reduz internações^(22,23)^.

Considerando a inexistência de instrumentos confiáveis que contemplassem as variáveis investigadas (atribuição, viabilidade e frequência), foi necessário proceder à verificação da consistência interna dos componentes da seção III do questionário como etapa inicial para assegurar a fidedignidade de dados obtidos. Observaram-se níveis satisfatórios de confiabilidade, aferidos pelo coeficiente alfa de Cronbach^(24)^, o que indica boa coerência entre os itens que compõem cada dimensão, demonstrando que o instrumento apresentou estabilidade adequada para mensurar os construtos avaliados. Embora os achados indiquem níveis satisfatórios de consistência interna, evidenciados pelos coeficientes alfa de Cronbach, é importante reconhecer que tais resultados não esgotam a avaliação psicométrica do instrumento. A análise realizada contempla apenas uma das dimensões da validade e confiabilidade, não incluindo, por exemplo, evidências de validade de construto (como análise fatorial) ou validade externa. Nesse sentido, ainda que os indicadores apontem para uma estrutura interna coerente, o instrumento deve ser compreendido como em fase inicial de desenvolvimento e com caráter exploratório.

A análise dos *clusters* evidenciou a existência de perfis distintos entre os enfermeiros da APS. Embora o *cluster* 1 concentre a maioria da amostra (78%), essa distribuição não indica baixa discriminação, mas a realidade do perfil epidemiológico da rede estudada. A análise identificou com precisão um grupo majoritário de “engajamento padrão” e, mais importante, isolou um subgrupo minoritário, agrupando-os no *cluster* 2. O subgrupo identificado no *cluster* 2 (22%) apresentou escores mais baixos, especialmente no componente II. Entretanto, sua interpretação requer cautela. O índice de Silhouette reduzido para esse *cluster* (0,10) indica baixa coesão interna e fraca separação em relação ao *cluster* 1, sugerindo que os indivíduos alocados nesse grupo não formam um conjunto claramente delimitado. Assim, esse *cluster* pode refletir, em parte, a reunião de casos que não se ajustaram adequadamente ao padrão predominante do *cluster* 1, e não necessariamente um grupo homogêneo com características próprias bem definidas.

Apesar de a análise de dados ter revelado que o *cluster* 2 não configura um grupo claramente definido em função da baixa coesão interna, seus resultados sinalizam a existência de um segmento de profissionais com maiores fragilidades percebidas, especialmente em dimensões centrais da prática. Isso sugere a necessidade de priorizar estratégias formativas estruturantes orientadas a esse conjunto de enfermeiros. Assim, mais do que sustentar categorias rígidas, os achados contribuem para orientar ações educativas direcionadas ao fortalecimento de competências essenciais, sobretudo entre aqueles que apresentam maior distanciamento do padrão predominante percebido.

Por outro lado, o *cluster* 1, embora apresente maior percepção de viabilidade, atribuição e frequência das práticas, também demonstrou níveis limitados de percepção sobre capacitação no componente II (49,8). Tal achado sugere possível desalinhamento entre confiança percebida e domínio técnico, que pode configurar o risco de “ilusão de competência”^(10,11,12)^.

Nesse sentido, os dois *clusters* não são apenas uma divisão de notas, mas a constatação de que a fragilidade em um ponto acaba afetando todo o desempenho do enfermeiro, o que corrobora a literatura que demonstra que atitudes mais favoráveis, maior domínio técnico e níveis elevados de autoeficácia tendem a favorecer o engajamento em práticas psicossociais e a incorporação de intervenções de saúde mental na APS^(25,26,27)^. Esses elementos, quando articulados ao suporte institucional, sugerem uma explicação para a maior propensão de profissionais desse grupo em realizar intervenções específicas e adotar estratégias de cuidado ampliado, de acordo com os achados dos estudos recentes.

Entre os componentes avaliados, o componente III (Intervenções educativas e gerenciais) foi o mais consolidado na percepção sobre a prática dos enfermeiros. Em contraste, o componente II (Intervenções específicas em saúde mental e psiquiatria) destacou-se como o mais desafiador para implementação na APS. Esse padrão identificado no *cluster* 1 é coerente com a literatura, que mostra que enfermeiros que se percebem mais preparados, com maior competência e com clareza de que a saúde mental integra suas atribuições tendem a se envolver mais ativamente em ações de cuidado psicossocial na APS. Estudos sobre práticas em saúde mental na APS apontam que a sensação de despreparo, o medo e a falta de qualificação específica funcionam como barreiras importantes para a oferta de intervenções, enquanto maior capacitação e apoio organizacional aumentam a resolutividade das equipes^(17,28)^.

A coesão entre as altas pontuações nos componentes I e III, que indicam reconhecimento e percepção de viabilidade das intervenções em saúde mental (com médias acima de 90), e o baixo nível de capacitação no componente II (48,6%) pode ser explicada pelas barreiras identificadas na literatura. A formação insuficiente em saúde mental na graduação, a sobrecarga de trabalho, a ausência de capacitações em serviço e a falta de integração curricular entre saúde mental e APS durante a formação^(29,30,31)^ comprometem a formação prática para esse componente. Além disso, análise recente das consultas de enfermagem em saúde mental mostrou lacunas nas competências de manejo, evidenciando necessidade de treinamento especializado para atuação qualificada^(32)^.

As pontuações médias do componente II, que contempla as percepções sobre intervenções mais específicas da saúde mental, como relacionamento terapêutico, elaboração de Projeto Terapêutico Singular, realização de grupos, uso de escalas e práticas de reabilitação psicossocial, obtiveram consistentemente pontuações inferiores aos dos componentes I e III em ambos os *clusters*. Esse resultado sugere que, na percepção dos participantes, as ações de organização e educação são mais facilmente implementadas, enquanto intervenções que exigem competências clínicas e relacionais complexas demandam formação adicional com conhecimentos específicos e competências relacionais complexas^(8,29)^, nem sempre contemplados na formação dos enfermeiros generalistas^(17,28,29,30,31)^. Esses achados reforçam a necessidade de fortalecer a formação em saúde mental na graduação em enfermagem, por meio da ampliação de experiências práticas, da inclusão de metodologias ativas e da integração ensino-serviço. Implicam, ainda, o desenvolvimento de programas de educação permanente com componentes práticos e supervisão clínica, bem como a implementação de políticas institucionais que priorizem rotinas e protocolos voltados ao cuidado psicossocial da população.

A combinação entre a formação deficiente e a ausência de educação permanente contribui para a manutenção de lacunas assistenciais, impactando negativamente a qualidade do cuidado. Mesmo diante da identificação das demandas em saúde mental, muitos profissionais demonstram dificuldades em aplicar conhecimentos básicos necessários à intervenção em saúde mental na APS^(30,33)^. Soma-se a isso a percepção, ainda comum entre os profissionais, de que essas práticas são exclusivas dos serviços especializados da RAPS, o que tende a restringir sua adoção no âmbito da APS^(34,35)^, contribuindo para os excessivos encaminhamentos^(16,21)^ e resultando na baixa procura por cuidados de saúde nesses espaços por parte das pessoas com TMGs e persistentes.

Entre as variáveis investigadas, observou-se associação significativa entre capacitação em dependência química e estar agrupado no *cluster* 1, sugerindo que enfermeiros que participaram em alguma capacitação em dependência química demonstraram maior confiança e habilidade para atuar em práticas psicossociais. Revisão sistemática^(36)^ evidenciou que treinamentos focados em transtornos por uso de substâncias melhoram significativamente o conhecimento técnico, as atitudes profissionais e a autoconfiança dos enfermeiros. Tal achado corrobora estudos que pontuam a capacitação como fator crítico para a qualificação da atenção à saúde mental na APS, sobretudo quando voltada para condições altamente estigmatizadas, como o uso problemático de substâncias psicoativas^(34,35,37,38)^.

A presença de profissionais mais jovens no *cluster* 1 sugere que tais profissionais possam estar mais disponíveis para a realização de intervenções em saúde mental no âmbito de suas práticas profissionais, além de estarem mais familiarizados com as diretrizes atuais da atenção psicossocial^(39)^. Devido ao tempo de formação, enfermeiros mais jovens tendem a ter acesso a currículos mais atualizados, o que favorece a adoção de práticas integradas e baseadas em evidências na APS. Além disso, a literatura demonstra o avanço nas discussões sobre as diretrizes curriculares de cursos de enfermagem com ênfase na necessidade de valorização dos conteúdos de saúde mental^(37,38,39)^.

Os resultados deste estudo reiteram a necessidade urgente de estratégias de educação permanente que contemplem, além do aprimoramento técnico, a especificidade do perfil profissional. Evidências recentes destacam que processos formativos eficazes devem incluir metodologias participativas, como oficinas clínicas, simulações realísticas, discussões de casos, supervisão matricial e educação interprofissional, favorecendo a integração teoria–prática e a reflexão crítica sobre o cuidado. No âmbito das competências relacionais, destacam-se habilidades como comunicação terapêutica, desenvolvimento da escuta qualificada, empatia, vínculo, negociação de planos de cuidado, tomada de decisão compartilhada e capacidade de atuar em situações de crise com postura acolhedora e não coercitiva. Essas competências são consideradas centrais para o desempenho do enfermeiro na identificação precoce de demandas em saúde mental, no estabelecimento de relações de confiança e na condução de intervenções clínicas baseadas em evidências em saúde mental^(7)^.

A partir dos resultados e de suas implicações práticas e políticas para a APS e para a formação em enfermagem em saúde mental, evidencia-se a necessidade de expandir e formalizar a consulta de enfermagem em saúde mental na APS, reconhecendo seu potencial para ampliar o acesso, fortalecer o vínculo e qualificar o cuidado psicossocial^(40)^. A integração sistemática da saúde mental à rotina da APS como eixo central da atenção integral demanda reorganização dos processos de trabalho e articulação efetiva entre os diferentes pontos da rede^(25)^.

Paralelamente, urge investir em programas de educação permanente que incluam componentes práticos, relacionais e de supervisão clínica, fundamentais para o desenvolvimento e a sustentação das competências necessárias à atuação dos enfermeiros nesse campo^(41)^. Tais medidas devem ser acompanhadas do reconhecimento formal da enfermagem como ator-chave na saúde mental comunitária, especialmente em países de baixa e média renda, onde há escassez de especialistas^(42,43)^. Ademais, destaca-se a valorização institucional da enfermagem em saúde mental como área legítima e estratégica de atuação, o que envolve condições adequadas de trabalho, apoio organizacional e visibilidade profissional. Por fim, os resultados apontam para a necessidade de políticas educacionais e regulatórias que fortaleçam a formação em saúde mental na graduação e padronizem competências essenciais para o exercício profissional, contribuindo para qualificar, de forma sustentável, a resposta da APS às demandas psicossociais.

### Limitações do Estudo

Os resultados deste estudo apontam achados relevantes sobre o papel do enfermeiro na APS e evidenciam lacunas persistentes em sua capacitação para intervenções específicas em saúde mental. Além disso, o estudo traz evidências sobre as percepções dos profissionais, bem como sobre as barreiras e potencialidades percebidas que estruturam sua prática clínica. Dessa forma, contribui para o avanço do conhecimento sobre os fatores que influenciam a identificação de demandas e a realização de intervenções em saúde mental no âmbito da APS, campo ainda pouco explorado na literatura nacional. Oferece evidências sobre percepções, barreiras e potencialidades da prática profissional em saúde mental nesse contexto, identificando grupos de profissionais com maior propensão às ações de saúde mental no contexto da APS. No entanto, algumas limitações devem ser consideradas quando se pensa na extrapolação dos resultados. Embora o instrumento de coleta tenha sido desenvolvido para identificar e descrever intervenções de enfermagem em saúde mental na APS, não foi concebido para mensurar um construto latente específico. Ele se baseia principalmente em normativas institucionais, evidências da literatura e avaliação de conteúdo por especialistas, buscando assegurar a pertinência das intervenções ao escopo da prática de enfermagem. Reconhece-se como limitação o fato de não terem sido conduzidas análises psicométricas mais robustas voltadas à avaliação da validade de construto e, por conseguinte, à fundamentação teórica formal. Estudos futuros poderão aprofundar a investigação das propriedades psicométricas do instrumento sugerido, incluindo análises fatoriais e outras abordagens de validação, de modo a ampliar a robustez metodológica da ferramenta. Além disso, não foram coletados dados relativos à prática e frequência de atendimento dos quadros de consumo prejudicial de substâncias psicoativas, o que restringe a amplitude da análise. Por se tratar de um delineamento transversal, não é possível estabelecer relações de causalidade entre as variáveis estudadas. A opção por oferecer um instrumento que possibilitasse maior agilidade em uma escala de três pontos pode ter incorrido em menor sensibilidade da escala para captar nuances e variações mais sutis nas percepções dos respondentes, o que pode reduzir a discriminação entre níveis intermediários de concordância ou capacitação. Além disso, destaca-se a baixa representatividade de certas categorias demográficas, como profissionais com 60 anos ou mais (n = 3), o que resultou em Intervalos de Confiança 95% amplos e baixo poder estatístico na regressão *log-binomial* para esta variável específica. No entanto, optou-se por manter tais estratos para preservar a fidedignidade da amostra levantada. Os achados referentes à faixa etária devem, portanto, ser interpretados como tendências indicativas que necessitam de confirmação em estudos com amostras maiores. A adesão parcial das UBSs pode introduzir viés de seleção, uma vez que as unidades participantes podem apresentar maior interesse, organização ou sensibilidade às temáticas de saúde mental em comparação às que não aderiram. Ademais, outra limitação do estudo refere-se ao possível viés de desejabilidade social, uma vez que as respostas foram autorreferidas pelos participantes. Nessa condição, os enfermeiros podem ter tendido a relatar práticas consideradas mais adequadas ou socialmente esperadas em seu contexto profissional. Embora o anonimato das respostas tenha sido assegurado, não é possível excluir completamente a influência desse tipo de viés nos resultados. Tais fatores podem comprometer a precisão das informações coletadas. Embora a amostra seja representativa de diferentes regiões de uma importante cidade brasileira, a generalização dos achados para outros contextos deve ser feita com prudência. Por fim, como limitação central, destaca-se que os achados se baseiam na autopercepção dos enfermeiros quanto à viabilidade, atribuição e capacitação para realizar intervenções, não permitindo inferir, de forma direta, sua efetiva execução na prática clínica. Essa possível dissociação entre percepção e prática real pode superestimar ou subestimar o desempenho profissional no contexto da APS. Assim, recomenda-se cautela na interpretação dos resultados, bem como a realização de estudos futuros que incluam medidas observacionais ou indicadores objetivos de prática assistencial.

## CONCLUSÃO

O fortalecimento das competências psicossociais dos enfermeiros é estratégico para qualificar, de forma sustentável, a resposta da APS às demandas em saúde mental. Os resultados indicam a necessidade de investir em formação específica, educação permanente e apoio institucional, especialmente direcionados aos diferentes perfis profissionais identificados.

## Data Availability

Todo o conjunto de dados que dá suporte aos resultados deste estudo foi publicado no próprio artigo.

# Translation: Intervenciones de salud mental realizadas por enfermeros en la Atención Primaria de Salud: análisis según el perfil profesional*

**DOI:** 10.1590/1980-220X-REEUSP-2025-0463es

**Authors:** Rafaela Sales Medeiros, Divane de Vargas, Jaqueline Lemos de Oliveira

**Affiliations:** 1 Universidade de São Paulo, Escola de Enfermagem, São Paulo, SP, Brasil.

**Keywords:** Enfermeras y Enfermeros, Atención a la Salud Mental, Atención Primaria de Salud

## Abstract

**Objetivo:**

Identificar las variables relacionadas con la percepción de los enfermeros sobre el reconocimiento de las necesidades de salud mental y la implementación de intervenciones en el contexto de la Atención Primaria de Salud.

**Método:**

Se realizó un estudio transversal con 239 enfermeras de Atención Primaria de Salud. Las intervenciones identificadas se agruparon en tres componentes: intervenciones universales; intervenciones específicas para la salud mental y la psiquiatría; e intervenciones educativas y de gestión. Los participantes evaluaron la viabilidad de la implementación, su relevancia para su ámbito de trabajo y su autopercepción de capacidad para realizar las intervenciones, respondiendo a un instrumento estructurado que fue revisado por expertos y sometido a una prueba piloto, la cual mostró una consistencia interna satisfactoria (α = 0,77–0,90). Se utilizó el análisis de clusters para identificar perfiles profesionales distintos en cuanto a la percepción de atribución, viabilidad, capacidad y frecuencia de implementación de las intervenciones. Posteriormente, se aplicó la regresión log-binomial para analizar la asociación entre las variables de interés y los clusters identificados.

**Resultados:**

Entre los participantes, el 48,4% informó brindar atención diaria a necesidades de salud mental, siendo la ansiedad la más frecuente (71,1%). El análisis de clusters indicó que los profesionales más jóvenes y aquellos con formación específica en dependencia química obtuvieron puntuaciones más altas en todos los componentes investigados. El componente relacionado con intervenciones específicas de salud mental obtuvo las puntuaciones y la percepción de competencia más bajas.

**Conclusión:**

Es necesario aumentar las inversiones en la formación y el desarrollo de capacidades de enfermería para llevar a cabo intervenciones de salud mental en la Atención Primaria de Salud.

## INTRODUCCIÓN

Los trastornos mentales constituyen un conjunto heterogéneo de afecciones que varían según la intensidad, el curso y el impacto funcional. La literatura ha diferenciado, de manera cada vez más precisa, los trastornos mentales comunes (TMC) de los trastornos mentales graves (TMG). Los TMC, como la depresión leve a moderada, la ansiedad y los trastornos somatoformes, presentan una alta prevalencia en la Atención Primaria de Salud (APS) y están asociados a un sufrimiento psíquico significativo, aunque generalmente no implican un deterioro grave de la autonomía ni una ruptura de los vínculos sociales. En contraste, los TGS, que incluyen la esquizofrenia, los trastornos psicóticos persistentes, el trastorno bipolar y los cuadros graves de uso o abuso de sustancias, exigen un seguimiento continuo, una mayor complejidad asistencial y una estrecha articulación con los servicios de la Red de Atención Psicosocial (RAP). Se estima que ocho de cada diez personas con TMG no reciben tratamiento^(1)^. La combinación de alta prevalencia y baja identificación refuerza la urgencia de respuestas estructuradas en el sistema de salud.

Ante lo expuesto, la integración de la salud mental en la APS se ha señalado como una estrategia clave para la reducción de estos problemas en las comunidades^(2)^. Por su capilaridad y proximidad a los territorios, la APS es capaz de ampliar el acceso, promover la detección temprana y ofrecer intervenciones oportunas para mitigar la invisibilidad y la falta de tratamiento de los problemas de salud mental. A pesar de ello, la integración de la atención de la salud mental en la APS aún enfrenta diversos desafíos, particularmente en contextos con escasez de recursos. La falta de profesionales capacitados, la financiación insuficiente y la fragilidad de la educación continua son barreras recurrentes. A esto se suma la persistente falta de concientización y de formación adecuada entre médicos, enfermeros y agentes comunitarios, lo que contribuye al estigma y la discriminación en torno a los trastornos mentales^(3,4)^.

Las acciones llevadas a cabo por personal no especializado pueden ampliar la capacidad de los servicios de salud mental en primera línea y mejorar significativamente la atención. En este contexto, especialmente en países de ingresos bajos y medios, como Brasil, donde el acceso a profesionales especializados es crítico, los enfermeros constituyen un recurso importante para hacer frente a estos problemas. Esto se debe a que los enfermeros de la APS suelen ser el primer punto de contacto de los usuarios con el sistema de salud. Más allá de los cuidados físicos, desempeñan un papel central en la atención de salud mental, especialmente en el establecimiento de vínculos, la escucha cualificada y la comunicación terapéutica. Estas dimensiones de la atención de salud mental permiten a los enfermeros desarrollar intervenciones para el manejo de crisis y la identificación temprana de señales de TMC y TMG. De esta manera, contribuyen no solo a mejorar la capacidad del servicio ante cuadros relacionados con la salud mental, sino también a la articulación con la RAPS^(5,6)^.

Sin embargo, a pesar del potencial de estos profesionales para enfrentar la problemática, aún encuentran dificultades para ampliar su ámbito de acción en salud mental en la APS^(7)^. La escasez de capacitación profesional, la escasa atención prestada al tema durante la formación^(8,9)^ y el estigma frente a la enfermedad mental^(3)^ contribuyen a la baja identificación y utilización de las intervenciones de enfermería en salud mental como una práctica inherente al escenario de la APS^(7)^.

A pesar de que son bien conocidas las barreras que encuentran los enfermeros para implementar acciones de salud mental en la APS^(7,8,9)^, con frecuencia estos hallazgos se investigan a partir de la percepción de los propios trabajadores. Sin embargo, la literatura señala que la percepción de competencia no siempre se corresponde con el desempeño efectivo en la práctica clínica. La teoría de la autoeficacia sostiene que la creencia del individuo en su capacidad para ejecutar determinadas acciones influye en su comportamiento y en la toma de decisiones; sin embargo, dicha percepción puede no reflejar necesariamente el nivel real de competencia o la calidad de la ejecución de las prácticas asistenciales^(10,11)^. En este sentido, estudios en el ámbito de la educación y las profesiones de la salud han discutido la existencia de una brecha entre la autopercepción de habilidades y el desempeño observado, denominada riesgo de “ilusión de competencia”^(10,11,12)^. Esto refuerza la importancia de enfoques basados en competencias que consideren no solo la confianza del profesional, sino también la movilización efectiva de conocimientos, habilidades y actitudes en la práctica clínica.

No se identificaron estudios publicados que permitan identificar perfiles distintos de enfermeros según características sociodemográficas, formativas y de capacitación que evidencien grupos con mayor o menor propensión a la incorporación de acciones de salud mental en su práctica cotidiana en la APS. Así, las investigaciones que permiten identificar posibles diferencias entre grupos específicos suponen un avance con respecto a estudios anteriores, que en general describen las prácticas de manera agregada, sin discriminar los patrones internos de heterogeneidad profesional. Un análisis que considere estos aspectos permite orientar estrategias formativas y de gestión más precisas, alineadas con las necesidades específicas del perfil profesional, fortaleciendo la institucionalización de la atención de enfermería en salud mental en la práctica de la APS.

Ante este escenario, resulta importante comprender los factores que influyen en la actuación del enfermero, considerando los aspectos formativos, institucionales y subjetivos que determinan su capacidad para identificar e implementar tales intervenciones. Con el fin de responder a la pregunta “¿Qué variables (sociodemográficas, formativas o institucionales) están asociadas a una mayor identificación y realización de intervenciones en salud mental por parte de los enfermeros que trabajan en la APS?”, este estudio tuvo como objetivo identificar las variables relacionadas con la percepción de estos profesionales a la hora de reconocer las demandas de salud mental e implementar intervenciones en el contexto de la APS.

## MÉTODO

### Diseño del Estudio

Se trata de un estudio transversal basado en la herramienta *STrengthening the Reporting of OBservational studies in Epidemiology*.

### Lugar y Período de la Recolección

Según la Secretaría Municipal de Salud de São Paulo, en el momento de la Recolección, el municipio contaba con 468 Unidades Básicas de Salud (UBS). De estas, 81 eran híbridas, con atención asociada a especialidades (consultorios médicos de especialidades), y fueron excluidas del estudio, lo que dio como resultado un total de 387 UBS tradicionales, las cuales se incluyeron en el estudio. Se contactó individualmente por correo electrónico y por teléfono a todos los gerentes de las posibles unidades participantes para aclarar los objetivos del estudio y solicitar autorización para realizar la Recolección. Al final, 66 (17 %) UBS aceptaron participar y conformaron el escenario de la Recolección de datos, que se llevó a cabo entre marzo y agosto de 2023.

### Muestra

La muestra estuvo compuesta por enfermeros que trabajaban en las unidades participantes del estudio. Los criterios de inclusión fueron: a) tener una relación laboral en la institución desde hacía, como mínimo, seis meses, tiempo considerado suficiente para garantizar la experiencia operativa en el territorio y la exposición a las rutinas de atención de la salud mental; y b) haber realizado al menos una atención en salud mental, entendida como cualquier consulta, acogida o derivación relacionada con quejas psíquicas, signos de sufrimiento mental, uso problemático de sustancias o seguimiento de personas con TMC o TMG. Teniendo en cuenta el número total de enfermeros vinculados a la Secretaría Municipal de Salud y asignados a las UBS involucradas en el momento de la Recolección (N = 403), se establecieron, para el cálculo de la muestra, un margen de error del 5 %, una estimación de rechazos y pérdidas del 5 %, la inclusión del 20 % para el control de posibles factores de confusión y un nivel de confianza del 95 %, lo que dio como resultado una muestra mínima estimada de 207 participantes. Al final de la recolección, 239 enfermeros cumplieron con los criterios y todos aceptaron participar en el estudio. Para asegurar la representatividad territorial y la precisión de las estimaciones, se adoptó un muestreo estratificado, proporcional al número de enfermeros por región administrativa. La distribución final de los participantes incluyó a ocho enfermeros de la región Central, 69 de la región Sur, 19 de la región Oeste, 38 de la región Norte y 73 de la región Este. Esta estrategia garantizó la inclusión equilibrada de los diferentes territorios y reflejó la heterogeneidad organizacional y demográfica de la red de APS del municipio de São Paulo.

### Recolección de Datos

Para la recolección de datos, se utilizó un cuestionario creado por los autores, que contenía 43 preguntas distribuidas en tres secciones: Sección I – con 16 variables que investigaban cuestiones relacionadas con el perfil, la formación académica y la formación específica en salud, además de variables sociodemográficas como sexo, edad, estado civil, caracterización de la institución formadora (pública, privada y otras), duración de la formación (en meses/años), preparación para el trabajo en salud mental y/o adicción química durante la licenciatura (se buscó identificar si los contenidos se impartieron predominantemente a través de clases expositivas, conferencias, seminarios, discusiones guiadas por el docente o actividades extracurriculares); Sección II: perfil de la formación específica en salud mental y atención en adicción química, que incluye siete variables que investigan si el profesional cuenta con posgrado y recibió capacitación en salud mental o en adicción química después de la licenciatura, y la frecuencia de atención de los principales cuadros de salud mental (depresión, ansiedad, esquizofrenia y síntomas somáticos de sufrimiento psíquico); Sección III: compuesta por 20 intervenciones de enfermería en salud mental en la APS, descritas en normativas y documentos técnicos nacionales y municipales, incluyendo los Cuadernos de Atención Básica del Ministerio de Salud, las Directrices de Atención Básica de la Secretaría Municipal de Salud de São Paulo^(13,14)^ y la Resolución COFEN n.º 599/2018^(15)^, que regulan la actuación de la enfermería en salud mental. Además, también se seleccionaron intervenciones descritas en una revisión de la evidencia sobre prácticas de enfermería en salud mental en la APS^(7)^. Estas intervenciones se agruparon, de acuerdo con las Directrices de Atención Primaria de la Secretaría Municipal de Salud de São Paulo^(14)^, en tres componentes: Componente I: Intervenciones de práctica universal, que abarcan intervenciones que interactúan con otras líneas de atención de la APS; Componente II: Intervenciones de práctica específica de salud mental y psiquiatría; y Componente III: Intervenciones basadas en actividades educativas y de gestión, relacionadas con la preparación del equipo de enfermería para actuar ante situaciones de salud mental y el registro (Cuadro 1).

**Cuadro 1:** Distribución de las intervenciones según la clasificación en los tres componentes de intervención – São Paulo, SP, Brasil, 2023.

Intervenciones del componente I	Intervenciones del componente II	Intervenciones del componente III
1. Acogida	3. Asesoramiento	16. Educación sanitaria
2. Triaje	5. Relación terapéutica	17. Formación continua
4. Consulta de enfermería	7. Seguridad del paciente relacionada con el uso de psicotrópicos	20. Registro de enfermería
6. Seguimiento prenatal	8. Derivaciones/apoyo matricial	
10. Atención a domicilio	9. Apoyo social	
15. Prácticas integradoras y complementarias	11. Apoyo familiar	
19. Gestión de casos	12. Proyecto terapéutico individual	
	13. Grupos terapéuticos	
	14. Aplicación de escalas	
	18. Prácticas avanzadas en rehabilitación psicosocial	

Aunque las intervenciones se extrajeron de fuentes normativas y científicas, que ya las reconocían como prácticas válidas y recomendadas en el ámbito nacional, se procedió a su evaluación por parte de jueces expertos con el objetivo de verificar la adecuación de la asignación de estas intervenciones en los tres componentes propuestos^(14)^. Para ello, participaron en el proceso cinco jueces, dos de ellos con experiencia en el área de APS y tres en el área de salud mental. Los especialistas analizaron la pertinencia y la adecuación de cada una de las 20 intervenciones en relación con los respectivos componentes, considerando también si dichas intervenciones estaban alineadas con el alcance de las acciones desarrolladas por enfermeros en los servicios de salud. Este procedimiento buscó asegurar una mayor coherencia entre las intervenciones y los componentes analíticos adoptados. A continuación, antes de la recolección definitiva de datos, se realizó una prueba piloto con la aplicación del instrumento a ocho enfermeros pertenecientes a la población objetivo del estudio. Este procedimiento tuvo como finalidad evaluar la claridad, la comprensión y la adecuación de las preguntas a las dimensiones propuestas. La prueba piloto permitió además verificar la consistencia de la estructura del cuestionario, el tiempo promedio de respuesta y la comprensión de los ítems y las escalas de respuesta por parte de los participantes, lo que permitió realizar ajustes puntuales antes de la aplicación final del instrumento. Los resultados de estas fases evidenciaron una buena comprensión de los ítems y la adecuación de la asignación de las intervenciones en los componentes propuestos, por lo que no fueron necesarias modificaciones adicionales antes de la aplicación final.

Para cada intervención enumerada en los tres componentes, se preguntó a los participantes sobre la frecuencia con la que realizaban la intervención en la práctica cotidiana, si la reconocían como una atribución del enfermero de APS y su percepción sobre su capacitación para ejecutarla. Para la creación de las nueve puntuaciones de los componentes I, II y III, se estableció una puntuación de 3 para las respuestas “sí”, 2 para “no estoy seguro” y 1 para “no” a las preguntas “¿Considera que esta intervención se puede realizar en la UBS?” y “En su opinión, ¿es esta una atribución del enfermero en la APS?”. Para la pregunta “¿Se siente capacitado para realizar esta intervención?”, se estableció una puntuación de 3 para las respuestas “totalmente”, 2 para “parcialmente” y 1 para “incapaz”. Teniendo en cuenta que la recolección de datos se llevó a cabo durante el horario laboral de los enfermeros, se optó por una escala de Likert de tres puntos, con el fin de facilitar la comprensión, reducir el tiempo de respuesta y minimizar las ambigüedades, además de favorecer una mayor consistencia en las respuestas. Para la interpretación, las puntuaciones de cada pregunta, considerando cada componente, se calcularon de manera que generaran una puntuación entre 0 y 100, siendo mejor cuanto mayor fuera la puntuación. Considerando las respuestas de cada ítem como una escala de Likert de 3 puntos, el proceso se inició con el cálculo de la puntuación bruta (PB) del instrumento. Esta EB se obtuvo a partir del promedio de las puntuaciones de los ítems de cada dimensión (Cuadro 1). Este cálculo se realizó utilizando la siguiente fórmula:


$$EB=\frac{q1+q2+\cdots +qn}{n}$$

A partir del cálculo del EB, se realizó la conversión lineal (puntuaciones de 0 a 100) según el siguiente cálculo:


$$Escore_{decadadimensão}=\left[\left(EB-1\right)/2\right]\times 100$$

Para la variable “frecuencia de atención”, se asignó una escala de puntuación de 0 a 3, según la regularidad indicada por el participante. La respuesta “nunca” recibió una puntuación de 0; “mensualmente” se puntuó con 1; “semanalmente” se puntuó con 2; y “diariamente” se puntuó con 3.

La recolección de datos se llevó a cabo mediante entrevistas presenciales realizadas por un equipo capacitado, compuesto por enfermeros con formación en salud mental y previamente capacitados en un manual estandarizado de procedimientos, con el fin de garantizar la uniformidad en el enfoque y la aplicación de los instrumentos. Inicialmente, se contactó a los gerentes de las UBS autorizadas para organizar la logística de la recolección y facilitar el acceso a los profesionales. El equipo de investigación se desplazó a las unidades en días y turnos previamente acordados con la dirección local. Se contactó a los enfermeros que trabajan en la APS en su propio lugar de trabajo, donde recibieron explicaciones sobre los objetivos del estudio, los criterios de participación, la confidencialidad y la duración de la entrevista. Aquellos que aceptaron participar fueron entrevistados en una sala reservada, garantizando la privacidad y la comodidad. Las entrevistas se llevaron a cabo de manera estandarizada, siguiendo rigurosamente el protocolo establecido, y los datos recopilados se ingresaron y almacenaron directamente en la plataforma REDCap.

### Análisis de Datos

Los datos se analizaron mediante estadística descriptiva. Para comparar las variables de interés en cuanto a las puntuaciones calculadas, se utilizaron pruebas no paramétricas de Mann-Whitney o Kruskall-Wallis, dependiendo del número de categorías de cada variable independiente. Para identificar perfiles distintos de enfermeros con las puntuaciones más altas, se realizó un análisis de *clúster* de tipo jerárquico, basado en las siguientes variables: sexo; grupo de edad; estado civil; formación en salud mental; formación en alcohol, drogas y salud; capacitación en dependencia química; y capacitación en salud mental. Las variables incluidas en el análisis de *clúster* se seleccionaron en función de su relevancia teórica y empírica para la caracterización de perfiles profesionales, representando el ciclo de competencia profesional: saber cuál es su función (atribución), disponer de los medios para ejecutarla (posibilidad), saber cómo ejecutarla (capacitación) y, por último, ejecutarla (frecuencia). Esta elección se basó en datos de la literatura, incluyendo variables señaladas como asociadas al fenómeno estudiado^(6,8,16,17)^. La validez y la estabilidad de la solución de *clúster* se garantizaron mediante un enfoque de convergencia de métodos. En primer lugar, se utilizó el parámetro *nstart* = 25 en el algoritmo *k-means* para asegurar que la solución final no representara un máximo local. La estructura de los *clústeres* se validó mediante el índice de Silhouette, que presentó un coeficiente promedio de 0,38, lo que confirma un *clúster* acorde con la literatura de estudios conductuales y de salud, en la que valores superiores a 0,3 son aceptables para justificar la existencia de grupos.

Aunque el *clúster* 1 presenta mayor cohesión (anchura media de 0,46) y el *clúster* 2, mayor heterogeneidad (0,10), el Análisis de Componentes Principales evidenció la separación total de los centroides sin superposición para k = 2, garantizando la estabilidad de la partición binaria (Figura 1). Por último, la validez aparente se demostró mediante la comparación visual de las medias, en la que los grupos presentaron diferencias relevantes en las 12 puntuaciones analizadas (Tabla 1).

**Figura 1:** Determinación de los *clústeres* para el análisis – São Paulo, SP, Brasil, 2023.

**Tabla 1:** Distribución de las respuestas de los participantes en las intervenciones por componente y dimensión en la Atención Primaria de Salud – São Paulo, SP, Brasil, 2023.

Intervenciones/componente	Dimensión	Sí (%)	No (%)
Intervenciones del componente I	Funciones del personal de enfermería en la Atención Primaria	90,4 %	9,6 %
	Viabilidad de su aplicación en el centro de atención primaria	91,3 %	8,7 %
	Percepción de la formación profesional	58,9 %	41,1 %
Intervenciones del componente II	Funciones del personal de enfermería en la Atención Primaria de Salud	84,1 %	15,9 %
	Viabilidad de su implementación en el centro de atención primaria	86,7 %	13,3 %
	Percepción de la formación profesional	48,6 %	51,4 %
Intervenciones del componente III	Funciones del personal de enfermería en la Atención Primaria de Salud	90,9 %	9,1 %
	Viabilidad de su implementación en el centro de atención primaria	92,6 %	7,4 %
	Percepción de la formación profesional	58,5 %	41,5 %

Para dar mayor fiabilidad a la interpretación de los resultados, se verificó la consistencia interna de los componentes que integraban la sección III del instrumento, relacionada con la frecuencia, la atribución y la capacitación. Para ello, se calculó el coeficiente alfa de Cronbach. Todos los análisis se realizaron en el *software* R, utilizando los paquetes “*cluster*” y “factoextra”. Para identificar la asociación entre las variables de interés y los clústeres, se utilizó un modelo de regresión *log-binomial* para estimar la razón de prevalencia^(18)^. Para todos los análisis, se adoptó un nivel de significación del 5 %.

### Consideraciones Éticas

El estudio fue aprobado por el Comité de Ética en Investigación de la institución sede, mediante el Dictamen n*.º*5.592.768. Todas las personas que aceptaron participar en la investigación recibieron una copia y firmaron el Término de Consentimiento Libre e Informado. Se explicaron los riesgos y beneficios, garantizando la confidencialidad a los participantes. Los datos se almacenaron en una base de datos encriptada, a la que solo tiene acceso el equipo de investigación.

## RESULTADOS

### Caracterización Sociodemográfica y Educativa de la Muestra

La muestra está compuesta, en su mayoría, por mujeres (88,2 %), con una edad media de 40,9 años (DE = 7,75), casadas (55,65 %), graduadas en instituciones privadas (82,43 %) y con una duración media de formación de 12 años (DE = 5,25). Llevan ejerciendo entre 5 y 10 años. En cuanto a la formación de posgrado, el 95,4 % poseía un posgrado *lato sensu*. En cuanto a la preparación recibida durante la formación en enfermería para trabajar en salud mental en la APS, el 75,2 % de los encuestados informó no haberla recibido, mientras que el 60,6 % también señaló no haber recibido preparación para trabajar con personas con necesidades relacionadas con el consumo de sustancias. Además, el 80 % de los entrevistados consideró que la licenciatura no los había preparado para esta actividad, y el 90 % identificó la insuficiencia de este contenido durante la licenciatura. La principal forma de capacitación mencionada por quienes indicaron haber recibido algún tipo de preparación durante la formación fue a través de clases y actividades extracurriculares. Cuando se les preguntó sobre la educación continua y la capacitación en el servicio, el 75,6 % y el 72,2 % de los encuestados informaron no haber participado en ninguna actividad de capacitación sobre el consumo de sustancias y la salud mental, respectivamente, en los últimos cinco años.

### Atribución, Viabilidad de Ejecución y Percepción de la Capacitación para Llevar a Cabo las Intervenciones en la Atención Primaria de Salud

En el análisis comparativo de los componentes, el mayor porcentaje de atribución se observó en el componente III (Intervenciones basadas en actividades educativas y de gestión) (90,9 %). La percepción de la viabilidad de la realización de la intervención en el centro de salud también fue más elevada en este componente (92,6 %). Por su parte, al analizar la percepción sobre la capacitación profesional, el componente I (Intervenciones de práctica universal), que abarca intervenciones que interactúan con otras líneas de atención de la APS, presentó una ligera ventaja (58,9 %) frente al resto. Por otro lado, los porcentajes más bajos en las tres dimensiones se registraron en el componente II (Intervenciones de práctica específica de salud mental y psiquiatría): 84,17 % (atribución del enfermero de APS), 86,7 % (viabilidad de realización en la APS) y 48,6 % (percepción de la capacitación).

### Frecuencia Percibida de la Atención de Casos de Salud Mental

Tras ser preguntados sobre la frecuencia de la atención de trastornos de salud mental, el 48,4 % de los profesionales indicaron que realizaban consultas diarias, el 28,4 % consultas semanales y el 20,9 % consultas mensuales. Al analizar las respuestas sobre la atención de las cuatro afecciones investigadas, se observó un predominio de la atención diaria para la ansiedad (71,1 %), la depresión (61,5 %) y el malestar difuso e inespecífico (54,8 %). Por su parte, los casos de esquizofrenia se atendían, en su mayoría, mensualmente (57,7 %).

### Fiabilidad de la Medida

En el análisis de consistencia interna de los componentes de la Sección III del instrumento, mediante el cálculo de los coeficientes alfa de Cronbach, se observó que los respectivos coeficientes (componente I = 0,79, componente II = 0,90 y componente III = 0,77) indicaban una buena consistencia interna de los conjuntos de intervenciones, lo que sugiere la fiabilidad de los componentes enumerados.

### Análisis de los Clústeres

El análisis por *clúster* dio como resultado dos perfiles. El *clúster* 1 agrupó a los profesionales con puntuaciones más altas en todos los componentes (Tabla 2).

**Tabla 2:** Distribución de las puntuaciones medias obtenidas por los participantes en los componentes I, II y III según las dimensiones evaluadas (Atribución del enfermero en la Atención Primaria de Salud; Posibilidad de realización en la Atención Primaria de Salud; y Percepción de capacitación y frecuencia de ejecución) y los *clústeres* identificados (n = 239) – São Paulo, SP, Brasil, 2023.

Variable	*Clúster*	
	1 (n = 187) Media (DE)	2 (n = 52) Media (DE)
Atribución relativa al componente I	96,2* (6,7)	77,1 (18,7)
Asignación correspondiente al componente II	92,3 (10,4)	60,2 (22,2)
Asignación correspondiente al componente III	98,2 (6,7)	69,9 (29)
Posibilidad de realizarse en relación con el componente I	97,0 (5,9)	80,6 (16,2)
Posibilidad de realizarse en relación con el componente II	93,5 (8,1)	68,2 (20,2)
Posibilidad de realizarse en relación con el componente III	98,0 (7,3)	77,6 (26,4)
Sentirse capacitado en relación con el componente I	78,6 (13,4)	54,0 (15,1)
Sentirse capacitado en relación con el componente II	71,2 (16,9)	43,8 (19,7)
Sentirse capacitado en relación con el componente III	80,7 (18,9)	51,3 (22,8)
Frecuencia de realización en relación con el componente I	64,6 (15,6)	51,0 (9,9)
Frecuencia de realización relativa al componente II	49,8 (19,9)	31,5 (13,9)
Frecuencia de realización relativa al componente III	54,6 (21,5)	40,6 (18,3)

DP: desviación estándar;

*Puntuación máxima posible: 100 puntos.

El análisis de regresión *log-binomial* indicó una asociación estadísticamente significativa entre la formación en adicciones y la distribución en *los clústeres*, lo que sugiere que los profesionales con formación en esta área son más frecuentes en *el clúster* 1. En cuanto al grupo de edad, los profesionales de hasta 59 años presentaron una menor prevalencia en *el clúster* 2, lo que indica un predominio de individuos más jóvenes en *el clúster* 1. Por otro lado, las variables sexo, estado civil, haber recibido formación en salud mental o en alcohol y otras drogas durante la carrera, así como la realización de formación en salud mental, no presentaron una asociación estadísticamente significativa con los *clústeres* identificados. En general, el *clúster* 1 está compuesto por enfermeros más jóvenes y con mayor cualificación específica para el cuidado de personas que consumen sustancias psicoactivas(Tabla 3).

**Tabla 3:** Distribución de las variables que mostraron una asociación significativa con los *clústeres* identificados (n = 239) – São Paulo, SP, Brasil, 2023.

Variable	*Clúster*		RP* (IC 95%) (grupo = 1)	Valor de p	RP (IC 95%) (grupo = 2)	Valor de p
	1 (n%)	2 (n%)				
Grupo de edad						
Hasta 44 (n = 160)	126 (78,7 %)	34 (21,2 %)	2,363 (0,348; 16,051)	0,544	**0,319 (0,115; 0,885)**	**0,024**
Entre 45 y 59 (n = 65)	52 (80 %)	13 (20 %)	2,4 (0,352; 16,356)	0,533	**0,3 (0,098; 0,919)**	**0,032**
60 o más (n = 3)**	1 (33,3 %)	2 (66,7 %)	ref.		ref.	
Formación en adicciones						
No (n = 180)	135 (75 %)	45 (25 %)	ref.		ref.	
Sí (n = 58)	51 (87,9 %)	7 (12,1 %)	**1,172 (1,032; 1,332)**	**0,014**	0,483 (0,231; 1,011)	**0,054**

*RP: razón de prevalencia estimada mediante un modelo de regresión log-binomial; IC 95%: intervalo de confianza del 95%;

**Las categorías con frecuencia absoluta reducida (n < 5) se mantuvieron para preservar la integridad de la muestra original, aunque los intervalos de confianza resultantes deben interpretarse con cautela debido a la menor precisión estadística.

## DISCUSIÓN

El objetivo del estudio fue identificar las variables relacionadas con la percepción de los enfermeros a la hora de reconocer las necesidades de salud mental y poner en práctica intervenciones en el contexto de la atención primaria. Se observó que el componente II (Intervenciones específicas en salud mental) presentó el nivel más bajo de autopercepción de capacitación entre los profesionales (48,6 %). Aun así, la mayoría de ellos evaluó estas intervenciones como viables (86,7 %) y compatibles con sus responsabilidades profesionales (84,1 %). El análisis de la frecuencia percibida por los entrevistados en la atención de casos de salud mental evidenció el predominio de los trastornos mentales comunes (TMC), como la ansiedad, la depresión y el sufrimiento psíquico, a menudo mencionados como parte de la atención diaria de los enfermeros, lo que indica una alta demanda y presencia de estos trastornos en la rutina de la atención de salud de la APS^(2,19)^. Por el contrario, los casos de trastornos mentales graves y persistentes, como la esquizofrenia, se reportaron como menos frecuentes, con atenciones mensuales, lo que puede estar relacionado con una mayor prevalencia de los trastornos mentales comunes en la población en comparación con los trastornos mentales graves (0,3 % a 1 %)^(20)^.

Aún en este contexto, la baja prevalencia de estos cuadros en la APS puede estar relacionada, entre otros factores, con la práctica recurrente de derivaciones directas a la RAPS^(16,21)^, especialmente a los Centros de Atención Psicosocial, fenómeno que puede contribuir a la baja prevalencia de esta población que busca atención de salud en la APS. Este fenómeno puede desincentivar la búsqueda de atención de salud en la APS, a pesar de que se considera un espacio estratégico para la atención de los TMG, especialmente por el monitoreo clínico, el apoyo a la adherencia y la articulación con la red, lo que favorece la continuidad de la atención y reduce las hospitalizaciones^(22,23)^.

Dada la inexistencia de instrumentos confiables que contemplaran las variables investigadas (atribución, viabilidad y frecuencia), fue necesario verificar la consistencia interna de los componentes de la sección III del cuestionario como etapa inicial para asegurar la fiabilidad de los datos obtenidos. Se observaron niveles satisfactorios de confiabilidad, medidos mediante el coeficiente alfa de Cronbach^(24)^, lo que indica una buena coherencia entre los ítems que componen cada dimensión, demostrando que el instrumento presentó una estabilidad adecuada para medir los constructos evaluados. Aunque los hallazgos indican niveles satisfactorios de consistencia interna, evidenciados por los coeficientes alfa de Cronbach, es importante reconocer que dichos resultados no agotan la evaluación psicométrica del instrumento. El análisis realizado contempla solo una de las dimensiones de la validez y la confiabilidad, sin incluir, por ejemplo, evidencias de validez de constructo (como el análisis factorial) o validez externa. En este sentido, aunque los indicadores apuntan a una estructura interna coherente, el instrumento debe entenderse como en una fase inicial de desarrollo y de carácter exploratorio.

El análisis de los *clústeres* evidenció la existencia de perfiles distintos entre los enfermeros de la APS. Aunque el *clúster* 1 concentra la mayor parte de la muestra (78 %), esta distribución no indica una baja discriminación, sino la realidad del perfil epidemiológico de la red estudiada. El análisis identificó con precisión un grupo mayoritario de “compromiso estándar” y, lo que es más importante, aisló un subgrupo minoritario, agrupándolo en el *clúster* 2. El subgrupo identificado en el *clúster* 2 (22 %) presentó puntuaciones más bajas, especialmente en el componente II. Sin embargo, su interpretación requiere cautela. El índice de Silhouette reducido para este *clúster* (0,10) indica una baja cohesión interna y una separación débil con respecto al *clúster* 1, lo que sugiere que los individuos asignados a este grupo no forman un conjunto claramente delimitado. Por lo tanto, este *clúster* puede reflejar, en parte, la reunión de casos que no se ajustaron adecuadamente al patrón predominante del *clúster* 1, y no necesariamente un grupo homogéneo con características propias bien definidas.

Aunque el análisis de datos reveló que el *clúster* 2 no constituye un grupo claramente definido debido a su baja cohesión interna, sus resultados señalan la existencia de un segmento de profesionales con mayores debilidades percibidas, especialmente en dimensiones centrales de la práctica. Esto sugiere la necesidad de priorizar estrategias formativas estructurantes orientadas a este conjunto de enfermeros. Así, más que mantener categorías rígidas, los hallazgos contribuyen a orientar acciones educativas dirigidas al fortalecimiento de competencias esenciales, sobre todo entre aquellos que presentan un mayor distanciamiento del patrón predominante percibido.

Por otro lado, el *clúster* 1, aunque presenta una mayor percepción de viabilidad, atribución y frecuencia de las prácticas, también demostró niveles limitados de percepción sobre la capacitación en el componente II (49,8). Este hallazgo sugiere una posible desalineación entre la confianza percibida y el dominio técnico, lo que puede configurar el riesgo de una “ilusión de competencia”^(10,11,12)^.

En este sentido, los dos *clústeres* no son solo una división de calificaciones, sino la constatación de que la fragilidad en un punto termina afectando todo el desempeño del enfermero, lo que corrobora la literatura que demuestra que actitudes más favorables, mayor dominio técnico y niveles elevados de autoeficacia tienden a favorecer el compromiso con prácticas psicosociales y la incorporación de intervenciones de salud mental en la APS^(25,26,27)^. Estos elementos, cuando se articulan con el apoyo institucional, sugieren una explicación para la mayor propensión de los profesionales de este grupo a realizar intervenciones específicas y adoptar estrategias de atención ampliada, de acuerdo con los hallazgos de estudios recientes.

Entre los componentes evaluados, el componente III (Intervenciones educativas y de gestión) fue el más consolidado en la percepción sobre la práctica de los enfermeros. En contraste, el componente II (Intervenciones específicas en salud mental y psiquiatría) se destacó como el más difícil de implementar en la APS. Este patrón identificado en el *clúster* 1 es coherente con la literatura, que muestra que los enfermeros que se perciben más preparados, con mayor competencia y con claridad de que la salud mental forma parte de sus atribuciones tienden a involucrarse más activamente en acciones de atención psicosocial en la APS. Los estudios sobre prácticas en salud mental en la APS señalan que la sensación de falta de preparación, el miedo y la falta de cualificación específica actúan como barreras importantes para la oferta de intervenciones, mientras que una mayor capacitación y apoyo organizacional aumentan la capacidad de resolución de los equipos^(17,28)^.

La cohesión entre las altas puntuaciones en los componentes I y III, que indican el reconocimiento y la percepción de la viabilidad de las intervenciones en salud mental (con promedios superiores a 90), y el bajo nivel de capacitación en el componente II (48,6 %) puede explicarse por las barreras identificadas en la literatura. La formación insuficiente en salud mental en la carrera universitaria, la sobrecarga de trabajo, la ausencia de capacitación en el servicio y la falta de integración curricular entre la salud mental y la APS durante la formación^(29,30,31)^ comprometen la formación práctica para este componente. Además, un análisis reciente de las consultas de enfermería en salud mental mostró lagunas en las competencias de manejo, lo que evidencia la necesidad de capacitación especializada para una actuación calificada^(32)^.

Las puntuaciones medias del componente II, que abarca las percepciones sobre intervenciones más específicas de la salud mental, como la relación terapéutica, la elaboración del Proyecto Terapéutico Singular, la realización de grupos, el uso de escalas y las prácticas de rehabilitación psicosocial, obtuvieron sistemáticamente puntuaciones inferiores a las de los componentes I y III en ambos *clusters*. Este resultado sugiere que, en la percepción de los participantes, las acciones de organización y educación se implementan más fácilmente, mientras que las intervenciones que exigen competencias clínicas y relacionales complejas requieren formación adicional con conocimientos específicos y habilidades relacionales complejas^(8,29)^, que no siempre se contemplan en la formación de los enfermeros generalistas^(17,28,29,30,31)^. Estos hallazgos refuerzan la necesidad de fortalecer la formación en salud mental en la licenciatura en enfermería, mediante la ampliación de las experiencias prácticas, la inclusión de metodologías activas y la integración entre la enseñanza y el servicio. Implican, además, el desarrollo de programas de educación continua con componentes prácticos y supervisión clínica, así como la implementación de políticas institucionales que prioricen rutinas y protocolos orientados al cuidado psicosocial de la población.

La combinación de una formación deficiente y la ausencia de educación continua contribuye al mantenimiento de brechas asistenciales, lo que afecta negativamente la calidad de la atención. Incluso ante la identificación de las demandas en salud mental, muchos profesionales muestran dificultades para aplicar los conocimientos básicos necesarios para la intervención en salud mental en la APS^(30,33)^. A esto se suma la percepción, aún común entre los profesionales, de que estas prácticas son exclusivas de los servicios especializados de la RAPS, lo que tiende a restringir su adopción en el ámbito de la APS^(34,35)^, contribuyendo a las derivaciones excesivas^(16,21)^ y resultando en una baja demanda de atención de salud en estos espacios por parte de las personas con TMC y persistentes.

Entre las variables investigadas, se observó una asociación significativa entre la capacitación en adicción química y estar agrupado en el *cluster* 1, lo que sugiere que los enfermeros que participaron en alguna capacitación en adicción química demostraron mayor confianza y habilidad para actuar en prácticas psicosociales. Una revisión sistemática^(36)^ puso de manifiesto que las capacitaciones enfocadas en los trastornos por consumo de sustancias mejoran significativamente el conocimiento técnico, las actitudes profesionales y la autoconfianza de los enfermeros. Este hallazgo corrobora estudios que señalan la capacitación como un factor crítico para la calidad de la atención de salud mental en la APS, sobre todo cuando se enfoca en condiciones altamente estigmatizadas, como el uso problemático de sustancias psicoactivas^(34,35,37,38)^.

La presencia de profesionales más jóvenes en el *grupo* 1 sugiere que estos profesionales podrían estar más disponibles para realizar intervenciones de salud mental en el marco de sus prácticas profesionales, además de estar más familiarizados con las directrices actuales de la atención psicosocial^(39)^. Debido a su tiempo de formación, los enfermeros más jóvenes tienden a tener acceso a planes de estudio más actualizados, lo que favorece la adopción de prácticas integradas y basadas en la evidencia en la APS. Además, la literatura demuestra el avance en los debates sobre las directrices curriculares de los cursos de enfermería, con énfasis en la necesidad de valorar los contenidos de salud mental^(37,38,39)^.

Los resultados de este estudio reiteran la necesidad urgente de estrategias de educación continua que contemplen, además del perfeccionamiento técnico, la especificidad del perfil profesional. Evidencias recientes destacan que los procesos formativos eficaces deben incluir metodologías participativas, como talleres clínicos, simulaciones realistas, discusiones de casos, supervisión matricial y educación interprofesional, favoreciendo la integración teoría-práctica y la reflexión crítica sobre la atención. En el ámbito de las competencias relacionales, se destacan habilidades como la comunicación terapéutica, el desarrollo de la escucha cualificada, la empatía, el vínculo, la negociación de planes de atención, la toma de decisiones compartida y la capacidad de actuar en situaciones de crisis con una postura acogedora y no coercitiva. Estas competencias se consideran fundamentales para el desempeño del enfermero en la identificación temprana de las demandas de salud mental, en el establecimiento de relaciones de confianza y en la realización de intervenciones clínicas basadas en la evidencia en salud mental^(7)^.

A partir de los resultados y de sus implicaciones prácticas y políticas para la APS y para la formación en enfermería de salud mental, se pone de manifiesto la necesidad de ampliar y formalizar la consulta de enfermería de salud mental en la APS, reconociendo su potencial para ampliar el acceso, fortalecer el vínculo y mejorar la atención psicosocial^(40)^.

La integración sistemática de la salud mental en la rutina de la APS como eje central de la atención integral exige una reorganización de los procesos de trabajo y una articulación efectiva entre los diferentes puntos de la red^(25)^.

Paralelamente, es urgente invertir en programas de educación continua que incluyan componentes prácticos, relacionales y de supervisión clínica, fundamentales para el desarrollo y el mantenimiento de las competencias necesarias para el desempeño de los enfermeros en este campo^(41)^. Dichas medidas deben ir acompañadas del reconocimiento formal de la enfermería como actor clave en la salud mental comunitaria, especialmente en países de ingresos bajos y medios, donde hay escasez de especialistas^(42,43)^. Además, se destaca la valorización institucional de la enfermería en salud mental como área legítima y estratégica de actuación, lo que implica condiciones de trabajo adecuadas, apoyo organizacional y visibilidad profesional. Por último, los resultados apuntan a la necesidad de políticas educativas y normativas que fortalezcan la formación en salud mental en la educación superior y estandaricen las competencias esenciales para el ejercicio profesional, contribuyendo a calificar, de manera sostenible, la respuesta de la APS a las demandas psicosociales.

### Limitaciones del Estudio

Los resultados de este estudio aportan hallazgos relevantes sobre el papel del enfermero en la APS y evidencian lagunas persistentes en su capacitación para intervenciones específicas en salud mental. Además, el estudio aporta evidencia sobre las percepciones de los profesionales, así como sobre las barreras y potencialidades percibidas que estructuran su práctica clínica. De este modo, contribuye al avance del conocimiento sobre los factores que influyen en la identificación de demandas y la realización de intervenciones en salud mental en el ámbito de la APS, un campo aún poco explorado en la literatura nacional.

Ofrece evidencia sobre las percepciones, las barreras y las potencialidades de la práctica profesional en salud mental en este contexto, identificando grupos de profesionales con mayor propensión a las acciones de salud mental en el contexto de la APS. Sin embargo, deben tenerse en cuenta algunas limitaciones a la hora de extrapolar los resultados. Aunque el instrumento de Recolección se desarrolló para identificar y describir las intervenciones de enfermería en salud mental en la APS, no se concibió para medir un constructo latente específico. Se basa principalmente en normativas institucionales, evidencia de la literatura y evaluación de contenido por parte de expertos, con el fin de asegurar la pertinencia de las intervenciones al ámbito de la práctica de enfermería.

Se reconoce como limitación el hecho de que no se hayan realizado análisis psicométricos más sólidos orientados a la evaluación de la validez del constructo y, por consiguiente, a la fundamentación teórica formal. Estudios futuros podrían profundizar en la investigación de las propiedades psicométricas del instrumento sugerido, incluyendo análisis factoriales y otros enfoques de validación, con el fin de ampliar la solidez metodológica de la herramienta. Además, no se recopilaron datos relativos a la práctica y la frecuencia de la atención de los casos de consumo nocivo de sustancias psicoactivas, lo que restringe el alcance del análisis. Al tratarse de un diseño transversal, no es posible establecer relaciones de causalidad entre las variables estudiadas. La decisión de ofrecer un instrumento que permitiera una mayor agilidad en una escala de tres puntos puede haber dado lugar a una menor sensibilidad de la escala para captar matices y variaciones más sutiles en las percepciones de los encuestados, lo que puede reducir la discriminación entre niveles intermedios de concordancia o capacitación.

Además, cabe destacar la baja representatividad de ciertas categorías demográficas, como los profesionales de 60 años o más (n = 3), lo que dio lugar a amplios intervalos de confianza del 95 % y a un bajo poder estadístico en la regresión *log-binomial* para esta variable específica. No obstante, se optó por mantener dichos estratos para preservar la fiabilidad de la muestra obtenida. Los hallazgos relativos al grupo de edad deben, por lo tanto, interpretarse como tendencias indicativas que requieren confirmación en estudios con muestras más amplias. La participación parcial de las UBS puede introducir un sesgo de selección, ya que las unidades participantes pueden mostrar mayor interés, organización o sensibilidad hacia los temas de salud mental en comparación con las que no participaron. Además, otra limitación del estudio se refiere al posible sesgo de deseabilidad social, ya que las respuestas fueron autoinformadas por los participantes. En este contexto, los enfermeros pueden haber tendido a informar prácticas consideradas más adecuadas o socialmente esperadas en su contexto profesional.

Aunque se garantizó el anonimato de las respuestas, no es posible excluir completamente la influencia de este tipo de sesgo en los resultados. Tales factores pueden comprometer la precisión de la información recopilada. Aunque la muestra es representativa de diferentes regiones de una importante ciudad brasileña, la generalización de los hallazgos a otros contextos debe realizarse con cautela. Por último, como limitación central, cabe destacar que los hallazgos se basan en la autopercepción de los enfermeros respecto a la viabilidad, la atribución y la capacitación para realizar intervenciones, lo que no permite inferir, de manera directa, su ejecución efectiva en la práctica clínica. Esta posible disociación entre la percepción y la práctica real puede sobreestimar o subestimar el desempeño profesional en el contexto de la APS. Por lo tanto, se recomienda cautela en la interpretación de los resultados, así como la realización de estudios futuros que incluyan medidas observacionales o indicadores objetivos de la práctica asistencial.

## CONCLUSIÓN

El fortalecimiento de las competencias psicosociales de los enfermeros es estratégico para calificar, de manera sostenible, la respuesta de la APS a las demandas en salud mental. Los resultados indican la necesidad de invertir en formación específica, educación continua y apoyo institucional, especialmente dirigidos a los diferentes perfiles profesionales identificados.

## Data Availability

Todo el conjunto de datos que respalda los resultados de este estudio se publicó en el propio artículo.
